# Perioperative Difficult Conversations With Guardians of Pediatric Patients: A Simulation-Based Workshop for Anesthesiology Practitioners Using the VitalTalk Framework

**DOI:** 10.15766/mep_2374-8265.11616

**Published:** 2026-07-07

**Authors:** Mitchell L. Phillips, Frank Yanko, Julia H. Vermylen, Heather A. Ballard

**Affiliations:** 1 Assistant Professor, Department of Anesthesiology, Nemours Children's Health System; 2 Preliminary Intern, Department of Medicine, University of Chicago, Northshore University Health System; 3 Associate Professor, Northwestern University, Feinberg School of Medicine, Department of Medicine and Medical Education, Northwestern Memorial Hospital; 4 Associate Professor, Northwestern University, Feinberg School of Medicine, Department of Anesthesiology and Medical Education, Ann & Robert H. Lurie Children's Hospital of Chicago

**Keywords:** Anesthesiology, Nurse Anesthetists, Curriculum, Communication, Patient Satisfaction, Clinical/Procedural Skills Training, Simulation

## Abstract

**Introduction:**

Effective communication of adverse events is a critical skill for health care practitioners, particularly those in anesthesiology. Despite the importance of delivering bad news to guardians after complications, many anesthesia practitioners receive little to no formal training in this area. This curriculum aims to address this by introducing a learning activity that integrates the SPIKES and NURSE frameworks with simulated parent (SP) encounters.

**Methods:**

A randomized waitlist control design was used to evaluate this 3-hour simulation curriculum. Learners were randomly assigned to receive either immediate training in communication skills (a 1-hour didactic session on the SPIKES and NURSE framework, followed by SP deliberate practice) or delayed training after 1 month. Both groups completed a pretest, posttest, and delayed posttest. Communication skills were evaluated using a published checklist based on the SPIKES and NURSE frameworks. Course surveys were also administered to assess changes in learners’ self-reported confidence and overall course satisfaction with the training.

**Results:**

Twenty-one learners (15 attending anesthesiologists and 6 certified registered nurse anesthetists) participated in this educational activity. Both groups demonstrated significant improvements in checklist-scored communication skills and self-reported confidence following the simulation curriculum (*P* < .001). In the Early Intervention group, these improvements in simulated communication skills were sustained after a 1-month delay. Overall, course satisfaction was high, with a median score of 5 (IQR 5-5).

**Discussion:**

Our findings demonstrate that a structured curriculum incorporating the SPIKES and NURSE frameworks, combined with SP deliberate practice, enhances communication skills for difficult perioperative conversations with pediatric guardians.

## Educational Objectives

By the end of this activity, learners will be able to:

1. Apply SPIKES (situation, perception, invitation, knowledge, empathy, summary/next steps) to difficult perioperative conversations.
2. Apply NURSE (naming, understanding, respecting, supporting, exploring) statements to explore patients’ and families’ emotions.
3. Determine how to implement SPIKES/NURSE frameworks for disclosing serious news in their own clinical practice.

## Introduction

Complication rates in the pediatric operating room (OR) have been reported to vary widely from 0.3% to nearly 50%, depending on the definitions used and the populations studied.^1–4^ These perioperative events range from commonly experienced problems such as emergence agitation and postoperative nausea and vomiting to more severe adverse events such as medication administration errors and unplanned intensive care unit admission. While many complications can be mitigated through quality improvement efforts, they are not entirely preventable. Disclosing such events to the guardians of pediatric patients requires strong interpersonal communication skills to maintain trust and preserve the practitioner-family relationship. More so, this need for effective communication aligns with the Accreditation Council for Graduate Medical Education's core competencies, which emphasize interpersonal and communication skills for exchanging information and collaborating with patients, families, and health care professionals.^5^ Although anesthesiology training programs have incorporated simulated patients to teach the delivery of bad news in contexts such as obstetrics and adult perioperative care,^6,7^ there remains a gap in the perioperative education literature regarding curricula focused on disclosing adverse events to the guardians of pediatric patients.

Anesthesiologists play a crucial role in health care, involving sedative administration, pain management, and decision-making regarding surgical procedures. Effectively communicating these choices and events to guardians of pediatric patients is essential and has been widely recognized as an area of improvement across pediatrics.^8–10^ Previous curricula have primarily focused on delivering information to simulated parents (SPs) in a nonoperative settings.^11^ The literature consistently highlights several essential skills necessary for delivering bad news: empathy, active listening, and clarity in information delivery.^12,13^ Structured frameworks such as SPIKES (setting, perception, invitation, knowledge, empathy, summary/steps) and NURSE (naming, understanding, respecting, supporting, exploring) offer a practical approach for teaching and reinforcing these communication skills.^14,15^ While the SPIKES framework has long been utilized in palliative care and oncology settings, the application of this framework in perioperative settings is innovative and necessary. These skills are crucial to anesthesiologists’ practice but are not innate, so they must be effectively taught.

Our institution lacked a curriculum to develop communication skills for breaking bad news to guardians of pediatric patients in the perioperative setting, and we found limited published curricula addressing this need. To address this gap, a curriculum was developed to equip anesthesia trainees, attending physicians, and certified registered nurse anesthetists (CRNAs) with these essential skills. This intervention functions as a formative teaching experience, creating a low-stakes environment where learners can develop and practice these skills. We hypothesized that learners who participated in a simulation-based curriculum would demonstrate improved communication skills and self-reported confidence in disclosing adverse events to guardians of pediatric patients.

## Methods

### Study Design

Our study was a randomized waitlist control trial of a simulation-based curriculum designed to improve anesthesia practitioners’ communication skills when disclosing adverse intraoperative events to the guardians of pediatric patients. This curriculum utilized a waitlist control design with crossover (Figure). A randomized waitlist control design is utilized when learners are randomly assigned to either start a curriculum right away or wait and receive it later.^16^ This approach enables comparison between the intervention and waitlist groups while ensuring that all participants ultimately receive the curriculum. Primary measurements were obtained on 3 testing encounters: (1) pretest for both groups, (2) posttest, and (3) delayed posttest. The posttest was directly after training for the Early Intervention group and 1 month after the pretest for the Delayed Intervention group. The delayed posttest encounter was 1 month after training for the Early Intervention group and directly after training for the Delayed Intervention group.

**Figure.**
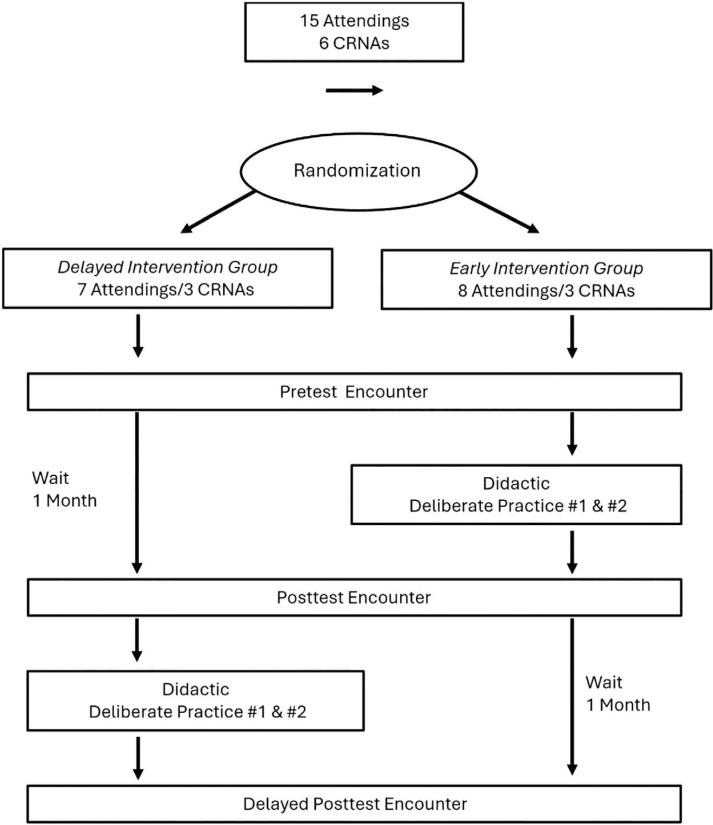
Randomized waitlist control study design. Abbreviation: CRNAs, certified registered nurse anesthetists.

### Procedure

Learners included attending physician anesthesiologists and CRNAs at Ann & Robert H. Lurie Children's Hospital of Chicago. The Ann & Robert H. Lurie Children's Institutional Review Board approved the study. Learners provided verbal consent and were randomly assigned to either the Early Intervention group or the waitlist control (Delayed Intervention) group (Figure). Both groups completed the pretest encounter following randomization. The Early Intervention group subsequently participated in an education curriculum that included a didactic component, deliberate practice with coaching, and a posttest encounter (posttest involving an SP). At 1 month, the Early Intervention group underwent a delayed posttest encounter. The Delayed Intervention group received no intervention during this month and continued routine clinical duties. One month after the pretest encounter, the Delayed Intervention group underwent a posttest encounter (prior to receiving any training), followed by the same education curriculum as the early group (didactic instruction, deliberate practice with coaching, and a delayed posttest encounter with an SP). All testing encounters were videorecorded and assessed using an adapted version of our published checklist.^17^ Trained raters were blinded to each learner's group or testing encounter (pretest, posttest, or delayed posttest).

### SP Recruitment and Training

SPs were recruited from the Northwestern University Simulation Center. To participate in simulation curricula, SPs completed a 2-hour orientation with the Simulation Center staff on resources, procedures, and expectations when interacting with undergraduate, graduate, and postlicensure health professionals.

Prior to implementation of the curriculum, our faculty conducted a 2-hour SP training session specifically focused on difficult conversations with pediatric guardians. SPs were emailed a handout outlining the parent role instructions (Appendix A) prior to training. During the training, faculty reviewed all 3 testing scenarios and 2 practice scenarios, providing the necessary medical background (ie, “What is a caudal nerve block?”) and guidance for preparing for their roles as parent guardians. Questions such as “What am I concerned about now?” or “What should my reaction to this situation be?” or “What is the expected outcome?” were addressed during this session. In addition, 1 faculty role-played the anesthesia practitioner while another provided feedback to the SP on their performance. SPs were encouraged to respond to the encounter how they best felt fit the situation given their assigned role. After each session, SPs were able to debrief with faculty SP educators.

### Pretest Encounter

Simulated parent interactions followed a consistent format across all testing encounters. The objective of the testing sessions was to simulate the disclosure of an adverse event to a guardian (played by the SP) of a pediatric patient undergoing surgery. Interactions took place in a room designed to resemble a family waiting area, equipped with chairs and a table for the learner and SP to sit across from each other. Learners were provided with a case stem outlining the patient's operative course and complication (Appendix B). After reviewing the case and asking clarifying questions, learners were expected to enter the room, disclose the adverse event, and conclude the conversation before exiting the room. Following each interaction, learners were provided time to debrief and ask questions with the faculty facilitator. These debriefs focused on emotional support only, and teaching was not performed at this time. All sessions were videorecorded for subsequent review and checklist scoring. In the pretest encounter, learners were tasked with disclosing that an infant had been admitted to the intensive care unit following an elective hernia repair due to a medication overdose (Appendix C; see Appendix B).

### Educational Activity

The learning activity, consisting of both a didactic session and deliberate practice, was designed to enhance skills in difficult perioperative conversations with guardians of pediatric patients. The curriculum began with a 1-hour didactic session focused on the SPIKES and NURSE frameworks, which emphasize strategies for breaking bad news and expressing empathy. This portion was delivered through a faculty-led slideshow presentation (Appendix D). Following the didactic session, learners participated in a small-group (2-3 students) deliberate practice session in which each student had the opportunity to practice delivering bad news to an SP. Deliberate practice scenarios 1 and 2 (Appendices E and F) were used with every group.^17^ All didactic lectures and deliberate practice sessions were led by Heather Ballard, a trained VitalTalk facilitator. VitalTalk is a program designed to empower health care practitioners with the skills to have difficult conversations surrounding health care decision-making.^18^ The VitalTalk framework provided the structure for deliberate practice in this curriculum by translating SPIKES and NURSE strategies into coachable communication behaviors. VitalTalk is a registered trademark, and we obtained permission to use and adapt the material for this activity. Institutions without VitalTalk-trained facilitators can implement a similar curriculum by teaching core SPIKES and NURSE techniques, using structured role-play with small-group coaching, and employing behaviorally anchored checklists (Appendix G) to guide feedback. The feedback and coaching portion of the deliberate practice followed a structured format. First, the learner reflected on their performance. This was followed by feedback from the facilitator and peers. The learner then identified a specific portion of the conversation where communication could be improved and chose how to revise their response. Finally, the learner “replayed” this segment of the conversation and reflected on the revised conversation and how to apply the communication strategies in clinical practice. The deliberate practice scenarios (see Appendices E and F) included disclosing multiple unsuccessful intravenous catheter attempts and discussing postoperative pain management after a failed caudal epidural nerve block.

### Posttest Encounter

The posttest encounter occurred after the educational activity for the Early Intervention group and 1 month after the pretest encounter for the Delayed Intervention group. In this scenario, learners were tasked with disclosing a medication error to the guardian that their child had received a medication to which they had a documented allergy (see Appendix B). The child does develop a rash after administration, but there are no long-term consequences (Appendix H). The structure of the encounter mirrored that of the pretest session, including the same time allotment, briefing, and debriefing procedures. All interactions were videorecorded for later review and evaluated using the same checklist as the baseline pretest encounter.

### Delayed Posttest Encounter

The delayed posttest encounter occurred 1 month after training for the Early Intervention group and directly after training for the Delayed Intervention group. This round of testing provided an opportunity to assess the Early Intervention group's ability to retain communication skills over time. Similarly to previous SP encounters, learners had to disclose serious news to an SP portraying the guardian of a pediatric patient undergoing surgery. In this scenario, the learner informed the SP that their child with kidney disease had inadvertently received a nephrotoxic medication (Appendix I; see Appendix B). Videorecording and checklist evaluation of this encounter was the same as the previous 2 testing interactions.

### Learner Assessment

After course completion, learners completed a survey reporting their years of clinical practice, prior experience with breaking bad news, and training in communication skills (Appendix J). A checklist (see Appendix G) was adapted from our previous work for perioperative difficult conversations using the SPIKES and NURSE frameworks.^17,19^ As a secondary outcome, course satisfaction and self-reported confidence before and after the educational activity were assessed using a 5-point Likert scale (1 = *very low*, 5 = *very high*).

The checklist was used for scoring all testing encounters (pretest, posttest, and delayed posttest) with SPs. The checklist used a dichotomous grading of 1 (done correctly) and 0 (not done/done correctly). All checklist components were weighted equally. Checklists were scored by 2 authors (Mitchell Phillips and Frank Yanko), who independently reviewed videorecordings of the testing encounters and scored the checklists. Both raters were blinded to group assignments and to pretest/posttest/delayed posttest status.

### Data Analysis

The primary outcome measures were changes in checklist scores from SP encounters. A secondary outcome measure was changes in confidence. Given the nonparametric nature of the data, Wilcoxon rank-sum tests were used to compare checklist scores and preintervention confidence to postintervention confidence levels. Stata version 15 (StataCorp, Austin, TX) was used to run all statistical analyses.

## Results

A total of 15 pediatric anesthesiology attendings and 6 CRNAs participated in the learning activity, all of whom verbally consented and completed the full curriculum. One hundred percent (100%) of learners completed the surveys. The median years of clinical experience were 10 (IQR 10-15) for attendings and 10 (IQR 7-11) for CRNAs. Most learners had prior experience in breaking bad news, with 100% of attendings and 83% of CRNAs reporting such experience. However, only 6 attendings (40%) and 2 CRNAs (33%) had received formal training in breaking bad news.

Table 1 shows checklist scores for learners grouped by Early Intervention and Delayed Intervention. Pretest encounter scores for both Early and Delayed Intervention groups were similar, with median scores of 11 (IQR 10-15) for the Early Intervention group and 11 (IQR 10-12) for the Delayed Intervention group (*P* = .91). Posttest encounter scores for the Early Intervention group, median score of 14 (IQR 14-16), were significantly higher than the Delayed Intervention group, median posttest score of 12 (IQR 12-13) (*P* < .001). After both groups were trained, delayed posttest encounter checklist scores were similar between the groups, with a median score of 15 (IQR 13-15) for the Early Intervention group and 15 (IQR 15.5-16) for the Delayed intervention group (*P* = .08). There was not a significant difference between the posttest for the Early Intervention group and the delayed posttest for the Delayed Intervention group (*P* = .30.)

**Table 1.**
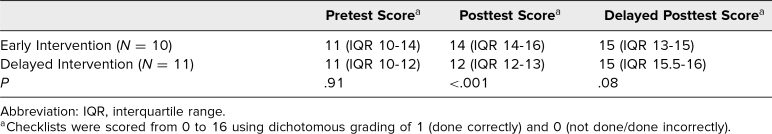
Checklist Scores Comparing Early Intervention and Delayed Intervention Groups

The course outcomes analyzed by assignment are shown in Table 2. Both the Early Intervention group (11 [IQR 10-14] to 15 [IQR 13-15], *P* = .001) and the Delayed Intervention group (11 [IQR 10-12] to 15 [IQR 15.5-16], *P* = .002) had significant increases in median checklist scores after training. In addition, both the Early Intervention group (2 [IQR 2-3] to 4 [IQR 4-4], *P* = .001) and the Delayed Intervention group (3 [IQR 3-3] to 4 [IQR 4-4], *P* = .008) had significant increases in self-reported confidence levels. There was a significant increase in checklist scores in the Early Intervention group between the posttest encounter and the delayed posttest encounter (*P* = .004), indicating that learning decay did not occur. Overall, course satisfaction was high in both groups (5 [IQR 4.5-5]).

**Table 2.**
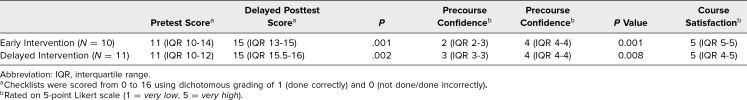
Curricular Outcomes Between Early and Delayed Intervention Groups (*N* = 21)

## Discussion

This difficult perioperative conversation curriculum improved anesthesia practitioners’ communication skills with pediatric guardians. By combining didactic instruction on the SPIKES and NURSE frameworks with deliberate practice through SP interactions, the learning activity significantly enhanced participants’ confidence and simulated skills in breaking bad news. The simulation-based approach provided a safe and structured environment for anesthesia practitioners to practice these challenging conversations and receive immediate feedback to reinforce learning. Overall, the educational intervention addressed a gap in training, as more than half of the participating attending physicians and CRNAs reported no prior formal education in communication skills despite routinely engaging in these conversations in clinical practice. Educators seeking to implement this curriculum could streamline the process by avoiding the multistep structure required for our randomized waitlist control study design. Instead, the curriculum may be delivered as a single 3-hour workshop that includes a formative pretest, didactic instruction, deliberate practice with standardized patients, and a posttest. Ultimately, this curriculum offers a scalable model for integration into anesthesiology training and continuing education programs, helping to ensure that practitioners are better equipped to navigate challenging conversations in the perioperative setting.

Beyond statistical significance, these findings demonstrate meaningful practical change, as learners showed observable improvement in key communication behaviors during simulated high-stakes perioperative conversations. The 3-point median increase in checklist scores reflects improvement across multiple discrete, behaviorally anchored skills aligned with the SPIKES and NURSE frameworks, including delivering a clear warning shot, eliciting guardian perception, and responding empathically. These gains indicate not only increased confidence but measurable changes in observable communication practices that are transferable to clinical care. The absence of significant differences between groups after both completed the intervention suggests that the curriculum reliably produces skill acquisition regardless of timing, and the sustained performance of the Early Intervention group supports short-term durability of learning. The parallel increase in self-reported confidence is particularly relevant in this context, as improved confidence may reduce avoidance of difficult conversations and promote more structured, empathic communication following adverse intraoperative events. This curriculum is readily adaptable to other institutions: educators can anchor teaching in established frameworks such as SPIKES and NURSE, use small-group deliberate practice to ensure active participation, employ a behaviorally anchored checklist for objective feedback, and incorporate structured debriefing to consolidate learning. Because the curriculum can be delivered in a half-day workshop format without requiring high-fidelity technology, it offers a feasible and scalable approach for programs seeking to strengthen perioperative communication skills with pediatric guardians.

In developing the curriculum, we recognized the importance of blending theoretical frameworks (SPIKES and NURSE) with practical application. Simulation has been shown to effectively improve skills in breaking bad news.^20,21^ The waitlist control design of the study allowed for randomization of groups and delayed assessment of pre- and postintervention within the same cohort.^16^ In our study design, learners were randomly assigned to either begin the intervention immediately or start it after a delay, allowing researchers to compare outcomes between the 2 groups during the waiting period. The advantage of this design is that it maintains the rigor of randomization while ensuring that all participants eventually receive the curriculum, which enhance ethical acceptability and participant recruitment. The SPIKES and NURSE framework development into a checklist enabled objective measurement of skill acquisition.^17^ Some aspects of the checklist items were more intuitive to our learners than others. For example, the checklist item of “sitting at an appropriate distance” was uniformly completed at baseline for learners. Other checklist components were more likely to be improved with training, such as giving a clear and concise “warning shot” and asking the SP about their perception of what was happening. Allowing opportunities to debrief allowed for feedback and facilitation of learning that has been shown to improve acquisition of skills in breaking bad news.^22^ One lesson learned from implementation of the curriculum was to consider the psychological impact that SPs experienced in portraying the parent. Negative psychological effects experienced by SPs after portraying emotional roles has been reported in the literature.^23,24^ To mitigate these effects on our SPs, we decided to change our scenarios from a death notification to breaking bad news over less morbid complications. Anecdotally, multiple SPs reported that portraying the parents of these lower-acuity scenarios felt less emotionally taxing compared to scenarios where the SP's child experienced a fatal complication.

This study has several limitations that should be considered. First, the sample size was small, which may limit the generalizability of the results; however, despite this, we observed statistically significant improvements in both checklist scores and self-reported confidence following training. Second, there were no trainees in this study due to the waitlist control design, as residents rotate at our pediatric hospital for 1–2 months only. In a previous curriculum, we trained pediatric anesthesiology fellows to have increased communication skills in an perioperative pediatric death notification scenario.^17^ Both undergraduate and graduate medical education students have been trained using the SPIKES and NURSE frameworks with significant improvement in simulated skills and confidence.^17,25^ Third, previous communication skills training may bias the results; however, the randomized waitlist control design helps limit this bias. Fourth, the clinical scenarios used in the simulations involved non–life-threatening complications, which may not fully reflect the complexity or emotional intensity of all pediatric anesthesiology cases. The scenarios were chosen purposefully, as our SPs thought it would be difficult to portray a parent receiving a death notification multiple times in a session. Although the study did include a retention component with a third encounter to assess skill retention, it is unclear whether the improvements in communication skills would persist over a longer period. Finally, other higher-level outcomes, such as behavior change in actual clinical encounters and family satisfaction, were not assessed and could provide more robust evidence of the curriculum's effectiveness.

This curriculum demonstrated that communication skills and confidence in breaking bad news can be improved through training with the SPIKES and NURSE frameworks. Future research should focus on a broader implementation of this curriculum among all perioperative practitioners who engage in difficult conversations with families. Expanding the range of simulation scenarios to include more complex, high-stakes clinical events may further enhance the training's applicability and better prepare practitioners for the emotional and cognitive demands of real-world encounters. Additionally, future studies should assess longer-term outcomes, such as changes in clinician behavior, retention of skills, and the impact on patient and family satisfaction.

## Appendices


SP Handout.docxLearner Case Stems.docxSP Case for Pretest.docxSlide Deck Didactic.pptxDeliberate Practice 1 Scenario.docxDeliberate Practice 2 Scenario.docxChecklist.docxSP Case for Posttest.docxSP Case for Delayed Posttest.docxPost Course Survey.docx
*All appendices are peer reviewed as integral parts of the Original Publication.*
