# Supplementary material for: Perioperative Difficult Conversations With Guardians of Pediatric Patients: A Simulation-Based Workshop for Anesthesiology Practitioners Using the VitalTalk Framework
Source: MedEdPORTAL. 2026 Jul 7;22:11616. doi: 10.15766/mep_2374-8265.11616 (PMC13337673; doi:10.15766/mep_2374-8265.11616)
Supplement: Supplementary file 1 — SP Handout.docxLearner Case Stems.docxSP Case for Pretest.docxSlide Deck Didactic.pptxDeliberate Practice 1 Scenario.docxDeliberate Practice 2 Scenario.docxChecklist.docxSP Case for Posttest.docxSP Case for Delayed Posttest.docxPost Course Survey.docx [file mep_2374-8265.11616-s001.zip › A. SP Handout.docx]

**Appendix A: Standardized Parent Handout**

This appendix contains the handouts provided to the standardized parents (SP) for all testing scenarios (pre-test, post-test, and delayed post-test) as well as for deliberate practice scenarios 1 and 2.

**Pre-test Scenario – Medication Error/Unexpected ICU Admission**

**Training Materials for Standardized Parent**

**Parent Profile**

*Age*:  30’s-40’s

*Gender*: male or female

*Race*: Same as SP

*Affect (Mannerisms, Behavior):* Exhibits concern and anxiety about his/her son's condition. Might be pacing, wringing hands, or showing other signs of stress. When spoken to, listens attentively, sometimes interrupting with questions or seeking reassurances. Might have a notebook and pen to take notes.

*Social History/Lifestyle*: Live in suburbs with husband/wife. First child.

*Occupation*: Lawyer

*Marital Status*:  Married

*General Appearance*: Business clothes, well kempt

**Scenario**

SP is a 30-40-year-old parent at the hospital for their child's planned inguinal hernia repair. Their child was born full term and is otherwise healthy. The parent is waiting for the practitioner to come into the room to hear about the surgery.

You are expecting the anesthesia practitioner to discuss the details of the surgery and tell you that you can go visit your child in the recovery room. Instead, the practitioner informs you that a medication error was made and that your child must go to the ICU with a breathing tube. Your confusion eventually changes to anger over the mistake.

**Instructions for Standardized Parent**

**How the SP responds to the practitioner’s entry.**

The parent should initially display a hopeful and expectant demeanor, anticipating positive news about their child's surgery. Upon hearing the practitioner’s entry, they might greet them politely but with a touch of anxiety, reflecting their concern for their child's well-being.

**The SP’s demeanor at the beginning and throughout the encounter (affect, nonverbal behavior)**

The parent's demeanor shifts from hopefulness to confusion, shown through a furrowed brow, widened eyes, and a slight gasp. As the explanation continues, the confusion turns to disbelief and then to anger, with the parent raising their voice and possibly gesturing emphatically.

**How the SP will respond to different interviewing styles**

*Empathetic and Calm:*

The parent will initially still be upset but will gradually calm down as they feel heard and understood. They might still express their frustration but will be more open to listening and asking questions rather than solely venting their anger. Nods occasionally, maintains eye contact, and might start to tear up as they express their fears and concerns.

*Authoritative and Direct:*

The parent's anger may escalate, feeling that their concerns are being dismissed or not fully acknowledged. They might interrupt the physician and demand more detailed explanations. Crosses arms, raises voice, and shows signs of agitation like tapping their foot or clenching their fists.

*Avoidant or Dismissive:*

The parent will become increasingly frustrated and might accuse the practitioner of being unprofessional or uncaring. They could threaten to file a complaint or take legal action. Leans forward aggressively, points fingers, and might refuse to listen further, insisting on speaking to someone higher up.

**Questions the SP will consistently ask during the encounter**

What exactly happened to my child?

How could such a mistake occur?

Is my child going to be okay?

How long will my child need to stay in the ICU?

What are you doing to ensure this doesn’t happen again?

Can I see my child now?

Who is responsible for this error?

**The challenges the SP will present to the practitioner**

*Emotional Reactivity:* The parent may shift rapidly from confusion to anger, making it challenging to maintain a calm and productive conversation.

*Trust Issues:* The parent might express a loss of trust in the medical team, questioning the competence of the staff involved.

*Demanding Detailed Explanations:* The parent will insist on thorough explanations and may not be satisfied with general responses, pressing for specifics about the error and the steps being taken to address it.

*Time Pressure:* The parent might demand immediate action and explanations, creating a sense of urgency and pressuring the anesthesia practitioner to provide quick but thorough responses.

*Potential Legal Threats:* The parent could hint at or explicitly mention taking legal action, adding another layer of stress to the encounter.

**Post-test Scenario – Allergic Reaction/Documented Allergy**

**Training Materials for Standardized Parent**

**Parent Profile**

*Age*:  30’s-40’s

*Gender*: male or female

*Race*: Same as SP

*Affect (Mannerisms, Behavior)*: Displays a blend of concern and protective behavior. When interacting with the practitioner, parent’s behavior is attentive but wary, with a tone that conveys worry and need for reassurance.

*Social History/Lifestyle*: Lives in city apartment with partner and two children

*Occupation*: Sales representative

*Marital Status*:  Married

*General Appearance*: Casual but tidy, wearing comfortable clothing suitable for a long day at the hospital.

**Scenario**

A 30-40-year-old parent is at the hospital after their 7-year-old child fell off their bike and fractured their arm. The child is otherwise healthy and was completely normal before the accident. The mother/father is nervous about their child undergoing surgery but did not expect anything bad to happen.

During the procedure, cefazolin was administered prior to the surgical incision, despite the child having a documented allergy to this medication. Shortly after the antibiotics were given, the child developed a rash consistent with an allergic reaction. There were no signs of anaphylaxis, a life-threatening allergic reaction.

You are in a waiting room, waiting for an update from the anesthesia team. When informed of your child’s reaction, you are initially in disbelief since this allergy was documented in their medical record. Eventually, your emotion changes to anger and fear.

**Instructions for Standardized Parent**

**How the SP responds to the practitioner’s entry.**

The parent will be anxious and on high alert, looking up immediately when the anesthesia practitioner enters the room. They might greet the practitioner with a mix of politeness and urgency, eager to hear about their child's condition.

**The SP’s demeanor at the beginning and throughout the encounter (affect, nonverbal behavior)**

Sitting on the edge of their seat, fidgeting with their hands, and making quick, nervous movements. Starts with nervousness, transitions to disbelief, and then to anger and fear Facial expressions show shock and disbelief when hearing about the medication error (wide eyes, mouth slightly open). As the conversation continues, the parent might start shaking their head, crossing their arms, and furrowing their brow. When anger sets in, they might lean forward, raise their voice, and use emphatic hand gestures.

**How the SP will respond to different interviewing styles**

*Empathetic and Calm:*

The parent will begin to feel heard and validated, which helps to gradually reduce their anger. They will still express their frustration and fear but will be more open to listening and engaging in a constructive dialogue. Calmer voice, occasional nodding, and more focused eye contact.

*Authoritative and Direct:*

The parent may become more defensive and escalate their anger, feeling that their concerns are not being fully acknowledged or respected. Interrupting the physician, raising their voice, and using more forceful gestures.

*Avoidant or Dismissive:*

The parent will likely become increasingly frustrated and may accuse the practitioner of being uncaring or unprofessional. They might demand to speak to a higher authority. Aggressive body language, such as leaning forward, pointing fingers, and possibly standing up in agitation.

**Questions the SP will consistently ask during the encounter**

How could this mistake happen when it’s clearly documented in his medical record?

Is my child going to be okay?

What are the immediate and long-term effects of this reaction?

How will you ensure this doesn’t happen again?

Who is responsible for this error?

**The challenges the SP will present to the practitioner**

*Emotional Reactivity:* The parent’s emotions will fluctuate between disbelief, anger, and fear, making it difficult to maintain a calm and productive conversation.

*Trust Issues:* The parent might express a loss of trust in the medical team, questioning the competence and reliability of the care provided.

*Demanding Accountability:* The parent will insist on understanding exactly how the error occurred and what specific measures are being taken to prevent future occurrences.

*Seeking Reassurances:* The parent will need constant reassurance about their child's current condition and the steps being taken to ensure their safety, requiring the physician to repeatedly affirm the protocols in place.

*Potential Legal Threats:* The parent could hint at or explicitly mention taking legal action, adding another layer of stress to the encounter.

**Delayed Post-test Scenario – Kidney Injury**

**Training Materials for Standardized Parent**

**Parent Profile**

*Age*:  30’s-40’s

*Gender*: male or female

*Race*: Same as SP

*Affect (Mannerisms, Behavior):* Shows a combination of worry and protectiveness. When interacting with the practitioner, parent’s behavior is attentive and cautious, with a tone that conveys deep concern and need for reassurance.

*Social History/Lifestyle:* Middle-class family living in an urban area. Parent is well-informed about Alexa's chronic kidney disease and take her medical condition seriously, ensuring she adheres to any dietary or lifestyle restrictions recommended by her healthcare providers.

*Occupation*: Full-time professional

*Marital Status:* Married

*General Appearance:* Business casual attire

**Scenario**

A 30-40-year-old parent is at the hospital with their 10-year-old child, who was brought in due to complaints of abdominal pain. At the hospital, the child was diagnosed with acute appendicitis and needed to undergo surgery to remove their appendix. The child has never been hospitalized before but frequently visits doctors because of their underlying kidney disease. They have been advised not to take any ibuprofen due to their kidney condition.

The surgery was uneventful, and the appendix was successfully removed. However, at the end of the procedure, the practitioner administered a medication called ketorolac, which is like ibuprofen—the medication your child has been told to avoid.

You are waiting alone in the waiting room. You have heard from the surgeon but are still waiting for an update from the anesthesia team. When informed of the medication error, you are initially in disbelief since this was documented in your child’s medical record. Eventually, your emotion changes to anger and fear of further kidney damage.

**Instructions for Standardized Parent**

**How the SP responds to the practitioner’s entry.**

The parent will look up immediately, displaying a mix of anticipation and anxiety. They might greet the practitioner with a polite but tense "Hello" or "Hi," their tone reflecting their eagerness to hear about their child's condition.

**The SP’s demeanor at the beginning and throughout the encounter (affect, nonverbal behavior)**

Anxious and on edge, with a noticeable worry about their child's well-being. Sitting upright, possibly fidgeting with their hands, and frequently glancing towards the door or the clock. Starts with nervous anticipation, shifts to disbelief upon hearing about the error, and then transitions to anger and fear. Initially, the parent might have wide eyes and a tense posture. When hearing about the medication error, they might lean back in shock, shake their head, or cover their mouth with their hand. As they become angry and fearful, they may lean forward, cross their arms, raise their voice, and use emphatic gestures like pointing or pounding a fist lightly on their knee.

**How the SP will respond to different interviewing styles**

*Empathetic and Calm:*

The parent will gradually calm down, feeling heard and understood. They will still express their anger and fear but will be more willing to engage in a constructive conversation. Calmer tone, occasional nodding, and more focused eye contact.

*Authoritative and Direct:*

The parent may become more defensive, and their anger might escalate, feeling that their concerns are not being fully acknowledged or respected. Interrupting the practitioner, raising their voice, and using more forceful gestures.

*Avoidant or Dismissive:*

The parent will likely become increasingly frustrated and might accuse the practitioner of being uncaring or unprofessional. They may demand to speak to someone higher up. Aggressive body language, such as leaning forward, pointing fingers, and possibly standing up in agitation.

**Questions the SP will consistently ask during the encounter**

How could this mistake happen when it’s clearly documented in my child’s medical record?

Is my child going to be okay?

What are the immediate and long-term effects on my child’s kidneys

What steps are you taking to monitor and protect my child now?

Who is responsible for this error?

**The challenges the SP will present to the practitioner**

*Emotional Reactivity:* The parent's emotions will fluctuate between disbelief, anger, and fear, making it challenging to maintain a calm and productive conversation.

*Trust Issues:* The parent might express a loss of trust in the medical team, questioning the competence and reliability of the care being provided.

*Demanding Accountability:* The parent will insist on understanding exactly how the error occurred and what specific measures are being taken to prevent future occurrences.

*Seeking Reassurance:* The parent will need constant reassurance about their child’s current condition and the steps being taken to ensure their safety, requiring the practitioner to repeatedly affirm the protocols in place.

*Potential Legal Threat:* The parent could hint at or explicitly mention taking legal action, adding another layer of stress to the encounter.

**Deliberate Practice Scenario #1 – Multiple IV Attempts**

**Training Materials for Standardized Parent**

**Parent Profile**

*Age*:  20’s-30’s

*Gender*: male or female

*Race*: Same as SP

*Affect (Mannerisms, Behavior):* Displays a combination of worry, protectiveness, and frustration. When interacting with the practitioner, parent’s mannerisms reflect concern and a need for answers.

*Social History/Lifestyle:* Lives in rural town with partner and two other children

*Occupation*: Stay at home mom or dad

*Marital Status:*Married, both parents present and actively involved in Arjun’s care and medical decisions

*General Appearance:* Casual clothes, well kempt

**Scenario**

The SP is a 20’s-30’s-year-old parent with a 2-month-old child who was scheduled for an MRI of the brain to evaluate for possible seizure activity. You were anxious about your child undergoing anesthesia. Although the anesthesiologist explained the risks of anesthesia and the possibility of multiple IV attempts, you did not expect it to result in so many bruises. You are particularly concerned about the bruise on your child’s forehead. You are upset and distressed over the appearance of your child and want to know what happened.

**Instructions for Standardized Parent**

**How the SP responds to the practitioner’s entry.**

The parent looks up immediately, their expression a mix of anxiety and anticipation. They may greet the practitioner with a polite but tense "Hello" or "Hi," reflecting their eagerness to hear about their child's condition and their concerns about the bruising.

**The SP’s demeanor at the beginning and throughout the encounter (affect, nonverbal behavior)**

The parent appears anxious and worried, with visible signs of distress. The initial worry transitions to disbelief and frustration as they learn more about the multiple IV attempts. Their tone may become more insistent and their expressions more intense. Initially attentive and anxious, the parent might furrow their brow, shake their head, or cover their mouth in disbelief. As frustration grows, they might lean forward, cross their arms, or use emphatic gestures like pointing or lightly tapping their knee.

**The SP’s concerns regarding his/her understanding of the situation**

The parent is particularly distressed by the bruise on their child’s forehead and is worried about the pain and potential trauma caused by multiple IV attempts. While they were informed about the risks of anesthesia and the possibility of multiple IV attempts, the extent of the bruising was unexpected and concerning. They want a clear explanation of what happened, why so many attempts were needed, and reassurance that their child will not suffer long-term effects from the bruising or the procedure.

**How the SP will respond to different interviewing styles**

*Empathetic and Calm:*

The parent will gradually calm down, feeling heard and understood. They will still express their frustration and concern but will be more willing to engage in a constructive conversation. Calmer tone, occasional nodding, and more focused eye contact.

*Authoritative and Direct:*

The parent may become more defensive, and their frustration might escalate, feeling that their concerns are not being fully acknowledged or respected. Interrupting the physician, raising their voice, and using more forceful gestures.

*Avoidant or Dismissive:*

The parent will likely become increasingly frustrated and might accuse the physician of being uncaring or unprofessional. They may demand to speak to someone higher up. Aggressive body language, such as leaning forward, pointing fingers, and possibly standing up in agitation.

**Questions the SP will consistently ask during the encounter**

Why did it take so many attempts to place the IV?

Couldn’t someone more experienced have done it?

Is this bruise going to cause any long-term issues?

How can we prevent this from happening again in the future?

Can you ensure my child wasn’t in too much pain?

What are the next steps in his care?

**The challenges the SP will present to the practitioner**

*Emotional Reactivity:* The parent’s emotions will fluctuate between anxiety, disbelief, frustration, and anger, making it challenging to maintain a calm and productive conversation.

*Trust Issues:* The parent might express a loss of trust in the medical team, questioning the competence and reliability of the care being provided. *Demanding Accountability:* The parent will insist on understanding exactly why the error occurred and what specific measures are being taken to prevent future occurrences. *Seeking Reassurances:* The parent will need constant reassurance about their child’s current condition and the steps being taken to ensure his safety, requiring the practitioner to repeatedly affirm the protocols in place.

*Potential Legal Threats:* The parent could hint at or explicitly mention taking legal action, adding another layer of stress to the encounter.

**Deliberate Practice Scenario #2 – Failed Caudal Nerve Block**

**Training Materials for Standardized Parent**

**Parent Profile**

*Age*: 30’s-40’s

*Gender*: male or female

*Race*: Same as SP

*Affect (Mannerisms, Behavior):* Exhibits a mix of concern and frustration.

*Social History/Lifestyle:* Middle-class family living in a suburban area.

*Occupation:* stay-at-home parent

*Marital Status:* Married

*General Appearance:* Dressed in casual but tidy clothing

**Scenario**

The SP is a 30-40-year-old parent with a 7-month-old child scheduled for a circumcision under general anesthesia. In the preoperative area, the anesthesiologist talked to you about performing a procedure called a caudal block to help with pain control. You were apprehensive about letting them perform the caudal block because it made you nervous for someone to put a needle so close to your child’s spine. However, you eventually agreed to let the anesthesiologist place the caudal block to help with pain control. Now that your child’s surgery is done, you are surprised to see your child in pain despite the caudal block. You are upset because you feel that you put your child at unnecessary risk without any benefit.

**Instructions for Standardized Parent**

**How the SP responds to the practitioner’s entry.**

The parent looks up immediately, their expression a mix of worry and frustration. They may greet the practitioner with a polite but tense "Hello" or "Hi," indicating their eagerness to address their concerns.

**The SP’s demeanor at the beginning and throughout the encounter (affect, nonverbal behavior)**

The parent appears anxious and concerned, with visible signs of distress. Sitting upright, possibly fidgeting, and maintaining a tense posture. The initial anxiety may transition to frustration and disappointment as they learn more about the caudal block’s ineffectiveness. Their tone may become more insistent, and their expressions more intense.

**How the SP will respond to different interviewing styles**

*Empathetic and Calm:*

The parent will gradually calm down, feeling heard and understood. They will still express their frustration and concern but will be more willing to engage in a constructive conversation. Calmer tone, occasional nodding, and more focused eye contact.

*Authoritative and Direct:*

The parent may become more defensive, and their frustration might escalate, feeling that their concerns are not being fully acknowledged or respected. Interrupting the practitioner, raising their voice, and using more forceful gestures.

*Avoidant or Dismissive:*

The parent will likely become increasingly frustrated and might accuse the physician of being uncaring or unprofessional. They may demand to speak to someone higher up. Aggressive body language, such as leaning forward, pointing fingers, and possibly standing up in agitation.

**Questions the SP will consistently ask during the encounter**

Why didn’t the caudal block work?

Couldn’t someone more experienced have done it?

Is it safe to give him more pain medication?

How can we ensure he won’t be in pain when we take him home?

What are the risks of the medications he’s receiving?

What are the next steps in his care?

**The challenges the SP will present to the practitioner**

*Emotional Reactivity:* The parent’s emotions will fluctuate between anxiety, disbelief, frustration, and anger, making it challenging to maintain a calm and productive conversation.

*Trust Issues:* The parent might express a loss of trust in the medical team, questioning the competence and reliability of the care being provided.

*Demanding Accountability:* The parent will insist on understanding exactly why the caudal block didn’t work and what specific measures are being taken to prevent future occurrences. *Seeking Reassurances:* The parent will need constant reassurance about their child’s current condition and the steps being taken to ensure their comfort and safety, requiring the practitioner to repeatedly affirm the protocols in place.

*Potential Legal Threats:* The parent could hint at or explicitly mention taking legal action, adding another layer of stress to the encounter.
