# Supplementary material for: Perioperative Difficult Conversations With Guardians of Pediatric Patients: A Simulation-Based Workshop for Anesthesiology Practitioners Using the VitalTalk Framework
Source: MedEdPORTAL. 2026 Jul 7;22:11616. doi: 10.15766/mep_2374-8265.11616 (PMC13337673; doi:10.15766/mep_2374-8265.11616)
Supplement: Supplementary file 1 — SP Handout.docxLearner Case Stems.docxSP Case for Pretest.docxSlide Deck Didactic.pptxDeliberate Practice 1 Scenario.docxDeliberate Practice 2 Scenario.docxChecklist.docxSP Case for Posttest.docxSP Case for Delayed Posttest.docxPost Course Survey.docx [file mep_2374-8265.11616-s001.zip › B. Learner Case Stems.docx]

**Appendix B: Learner Case Stems**

This appendix contains the case stems provided to learners for all testing scenarios (pre-test, post-test, and delayed post-test) as well as for deliberate practice scenarios 1 and 2.

**Pre-test Scenario – Medication Error/Unexpected ICU Admission**

Stem to provide to the learners:

*Willa Wolfe is a 7-month-old, 7 kg female who presents for bilateral laparoscopic inguinal herniorrhaphy under general anesthesia. She has no other significant medical history besides bilateral inguinal hernia. She was born full term and went home shortly after birth.*

*She underwent an uncomplicated anesthetic. The surgeon is almost done with the surgery, but comments that the patient seems inadequately relaxed. In a haste to administer muscle relaxant, you accidently administer 40 mg of rocuronium.*

*The surgery is now finished, and the patient has no twitches. Unfortunately, the location that you are practicing at does not have sugammadex. The surgeon, who has a full schedule, would like the patient taken to the ICU where they can wait until the muscle relaxant wears off.*

*The surgeon asks you to discuss the medication error with the family.*

**Post-test Scenario – Allergic Reaction/Documented Allergy**

Stem to provide to the learners:

*Carson Smith is a 7-year-old, 35 kg male with supracondylar fracture of left elbow who presents for closed reduction and percutaneous pinning under general anesthesia. His past medical history is significant for prematurity (born at 33 weeks) and a cefazolin allergy. He has been appropriately fasting, last having solids 8 hours ago. His left arm is splinted in a sling, and he has a 22g PIV in his right hand.*

*He underwent an uncomplicated induction. The surgeon is completing his time-out when they ask for perioperative antibiotics to be administered. In a haste to administer antibiotics, you administer cefazolin to the patient. Shortly after the patient develops diffuse urticaria. His hemodynamics otherwise remain stable, and you give diphenhydramine.*

*The remainder of the surgery is uncomplicated. The patient is now recovering in the post-anesthesia care unit (PACU), while the parent is waiting in a conference room to meet with the surgical team. The surgeon asks you to speak with the parent to explain the event that occurred …*

**Delayed Post-test Scenario – Kidney Injury**

Stem to provide to the learners:

*Alexa is a 10-year-old, 30 kg female who presents for laparoscopic appendectomy under general anesthesia. She has a medical history significant for chronic kidney disease.*

*She underwent an uncomplicated anesthetic. At the end of the surgery, you accidentally give a dose of ketorolac to help with pain control, forgetting about the history of chronic kidney injury. You are concerned about the risk of further kidney damage in the setting of ketorolac administration.*

*You decide to disclose the medication error to the family and discuss your concerns regarding the patient’s renal function.*

**Deliberate Practice Scenario #1 – Multiple IV Attempts**

Stem to provide to the learners:

*Arjun Patel is a 2-month-old, 6 kg male who presents for MRI brain with and without contrast to evaluate for possible seizures. He has no other significant medical history besides possible seizures. He was born full term and went home shortly after birth.*

*Induction of anesthesia was uncomplicated. You are working alone and the nurse in the room does not feel comfortable attempting an IV in a patient this young. You insert a supraglottic airway without any issue and begin to look for a vein. After five failed attempts you finally place a 24 g PIV in a scalp vein.*

*The rest of the anesthetic is uncomplicated. Shortly after the patient is taken to the PACU you receive a phone call from the nurse telling you that the family is really upset about the number of IV attempts and the decision to place an IV in the scalp. He now has a bruise on the side of his head.*

*The family is requesting to talk to you about what happened.*

**Deliberate Practice Scenario #2 – Failed Caudal Nerve Block**

Stem to provide to the learners:

*Andrew is a 7-month-old, 7 kg male who presents for hypospadias repair under general anesthesia. He has no other significant medical history. He was born full term and went home shortly after birth.*

*You are taking over the case for your colleague. During report, the anesthesiologist handing off the case reports that there was difficulty in placing the caudal and he is not sure that it is working. The patient has already required 20 mcg of fentanyl during the case.*

*The surgery is now finished, and the patient is in the PACU. The patient is fussy and the PACU nurse is requesting a dose of morphine for pain. The family would like to talk with you about the patient’s pain control and why the caudal doesn’t seem to be working.*
