# Supplementary material for: Perioperative Difficult Conversations With Guardians of Pediatric Patients: A Simulation-Based Workshop for Anesthesiology Practitioners Using the VitalTalk Framework
Source: MedEdPORTAL. 2026 Jul 7;22:11616. doi: 10.15766/mep_2374-8265.11616 (PMC13337673; doi:10.15766/mep_2374-8265.11616)
Supplement: Supplementary file 1 — SP Handout.docxLearner Case Stems.docxSP Case for Pretest.docxSlide Deck Didactic.pptxDeliberate Practice 1 Scenario.docxDeliberate Practice 2 Scenario.docxChecklist.docxSP Case for Posttest.docxSP Case for Delayed Posttest.docxPost Course Survey.docx [file mep_2374-8265.11616-s001.zip › D. Slide deck didactic.pptx]

## Slide 1
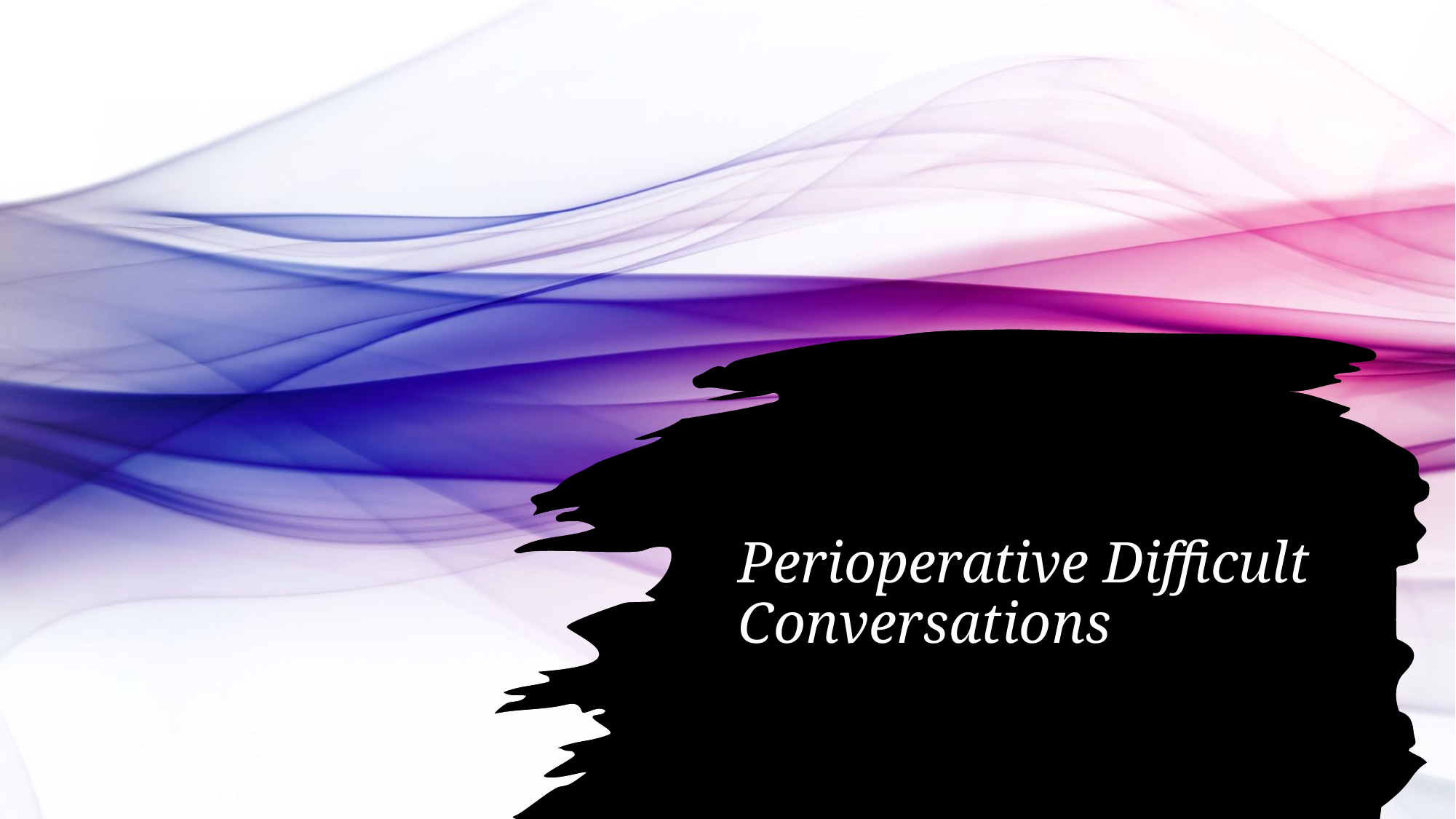

# Perioperative Difficult Conversations

## Slide 2
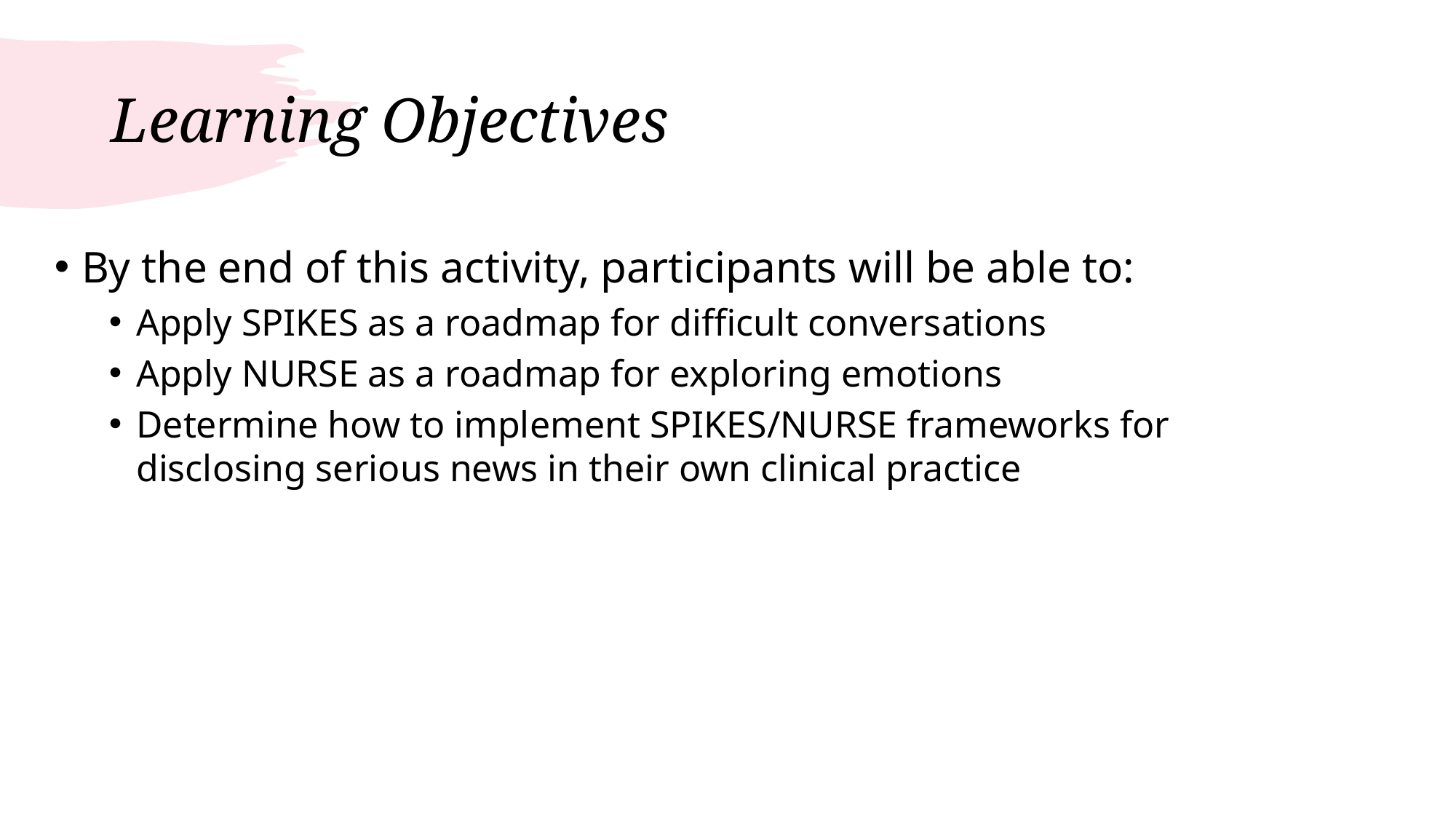

# Learning Objectives
By the end of this activity, participants will be able to:
Apply SPIKES as a roadmap for difficult conversations
Apply NURSE as a roadmap for exploring emotions
Determine how to implement SPIKES/NURSE frameworks for disclosing serious news in their own clinical practice

## Slide 3
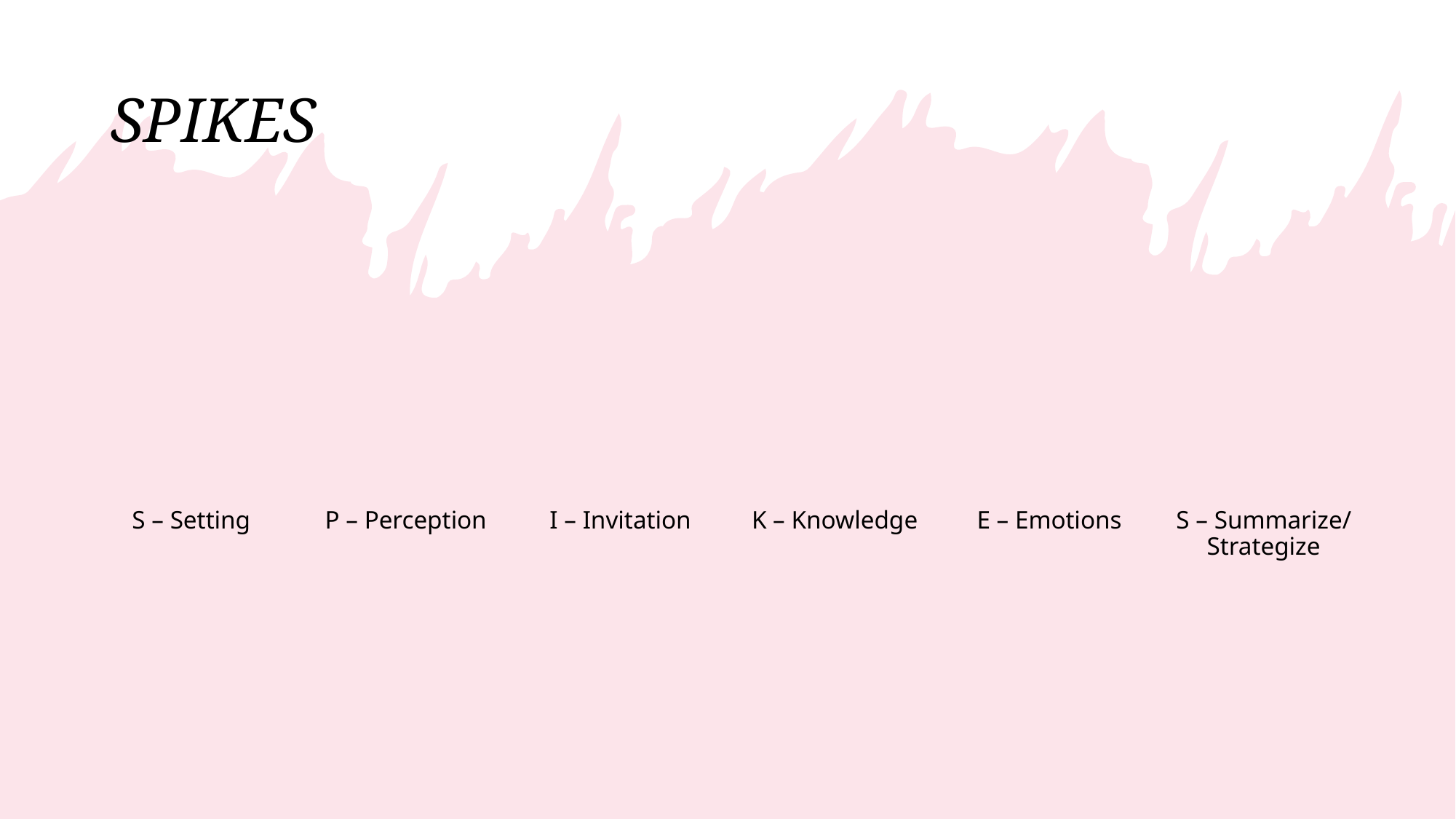

# SPIKES

## Slide 4
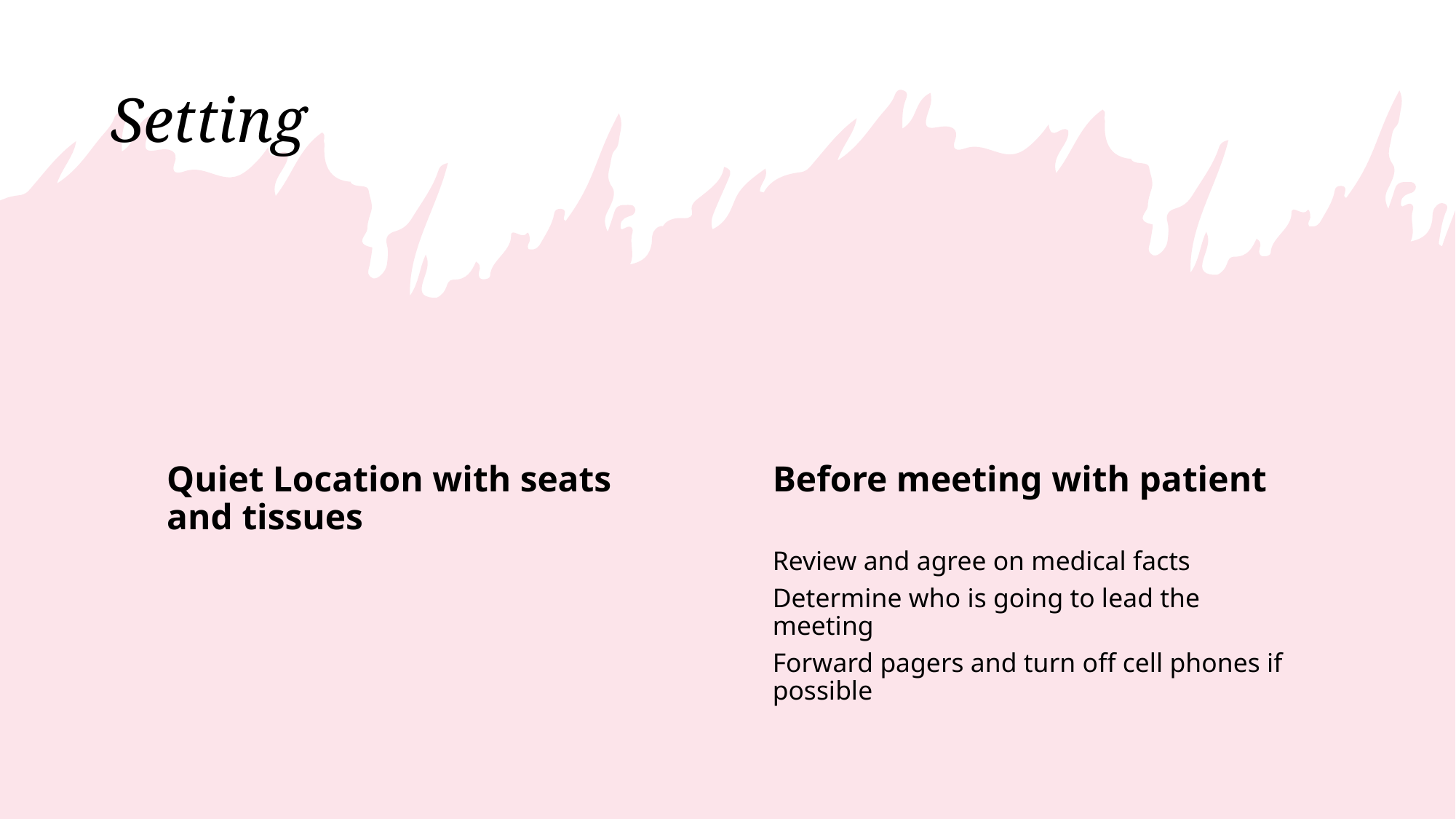

# Setting

## Slide 5
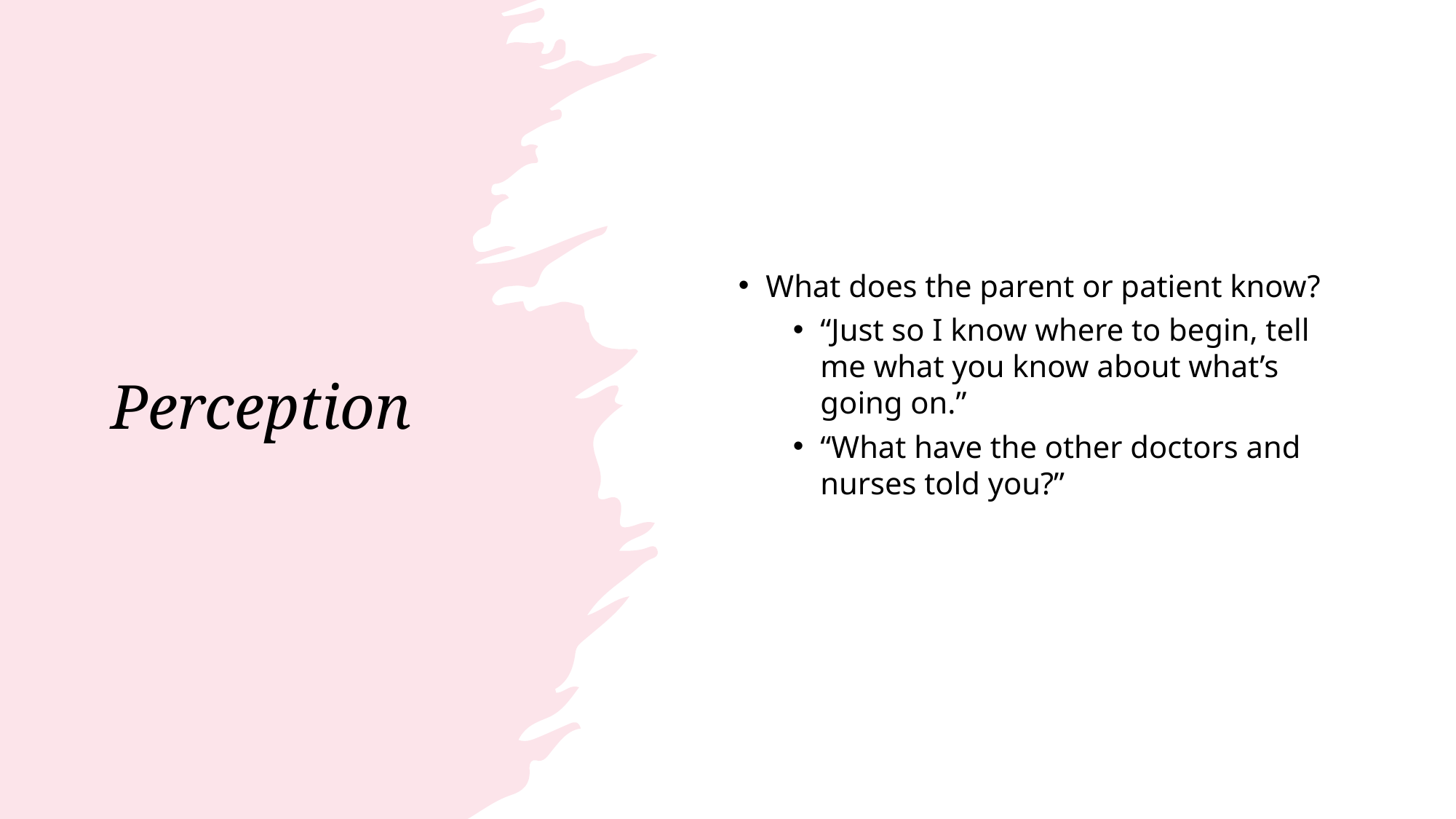

# Perception
What does the parent or patient know?
“Just so I know where to begin, tell me what you know about what’s going on.”
“What have the other doctors and nurses told you?”

## Slide 6
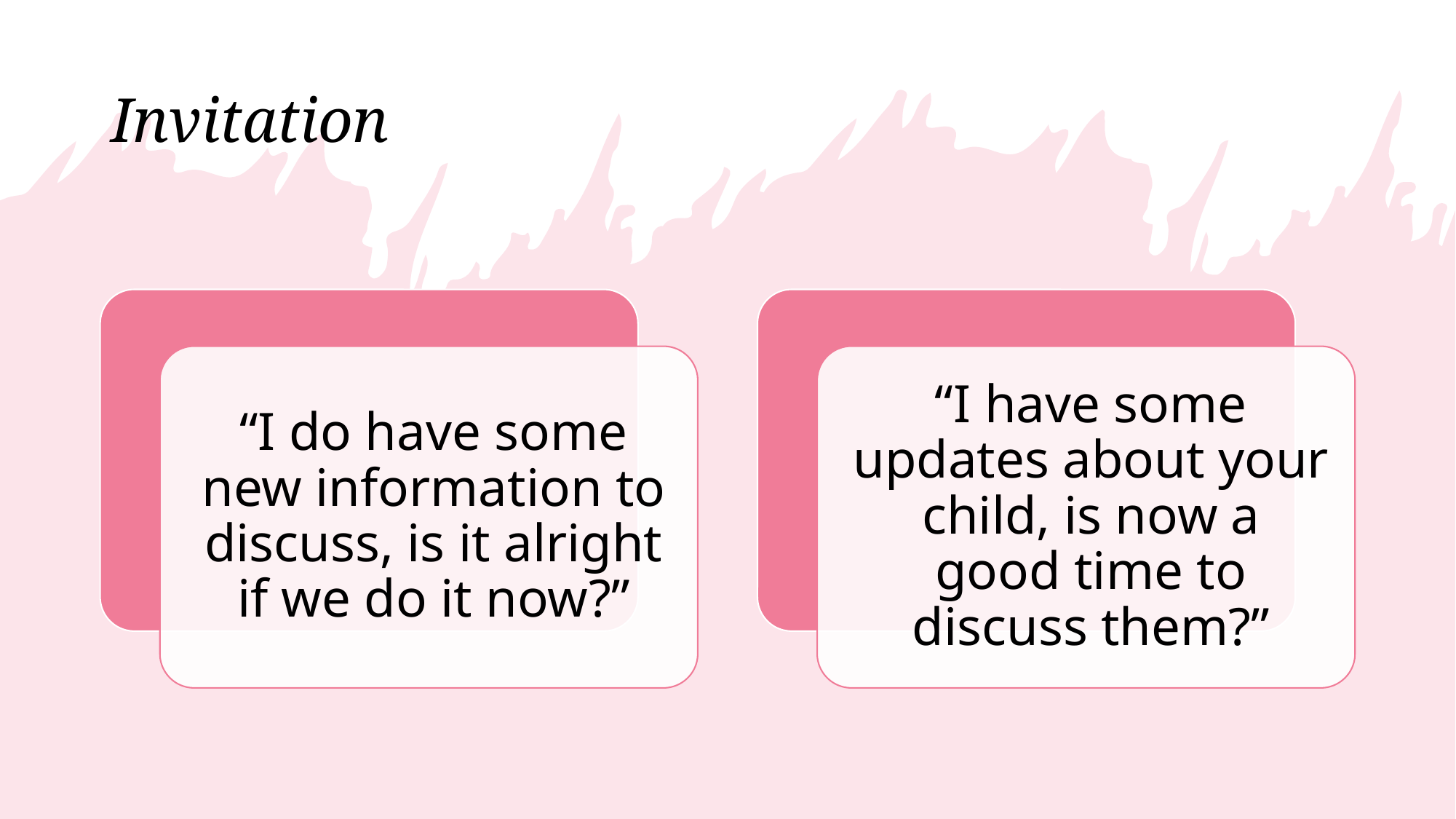

# Invitation

## Slide 7
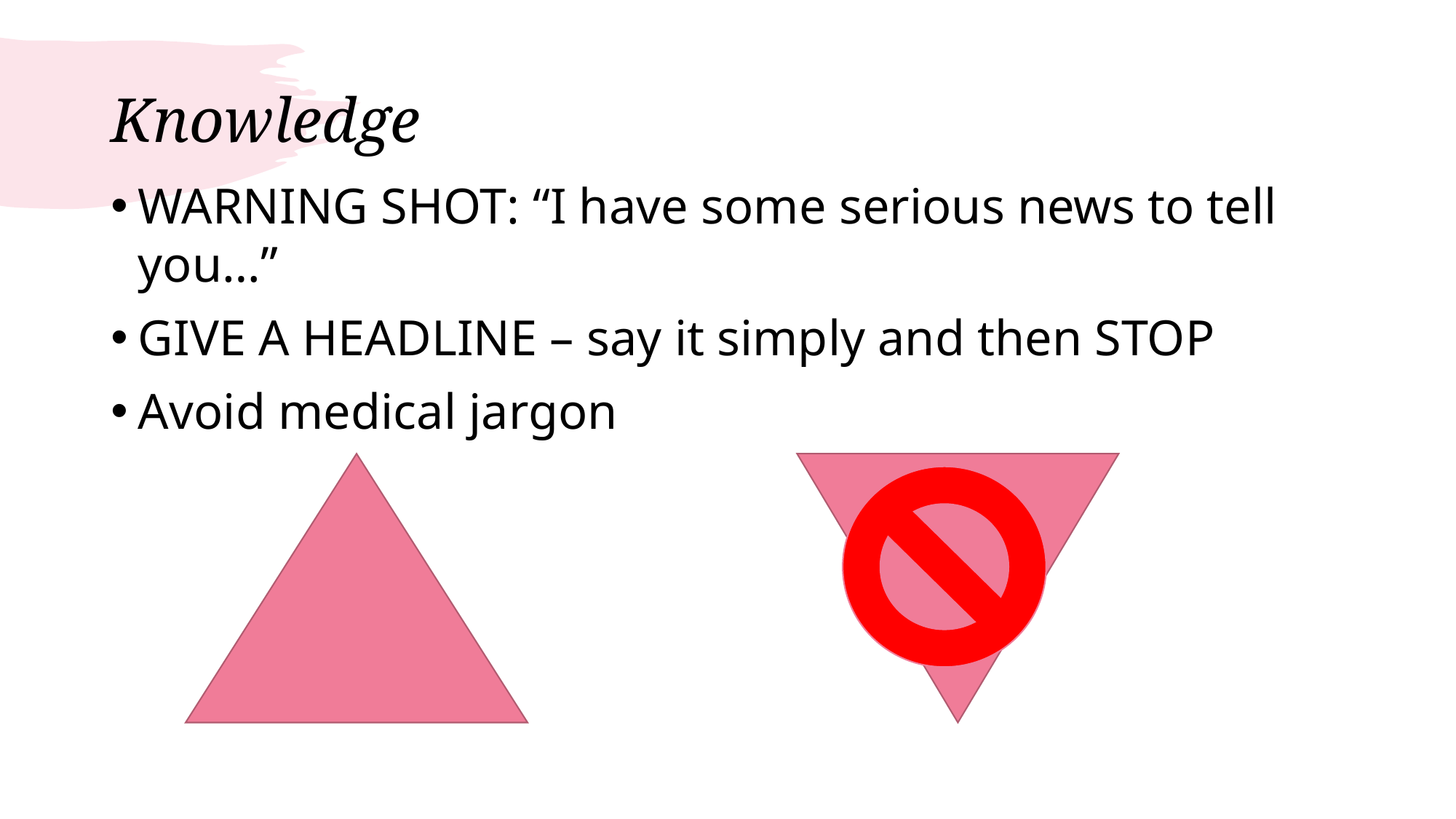

# Knowledge
WARNING SHOT: “I have some serious news to tell you…”
GIVE A HEADLINE – say it simply and then STOP
Avoid medical jargon

## Slide 8
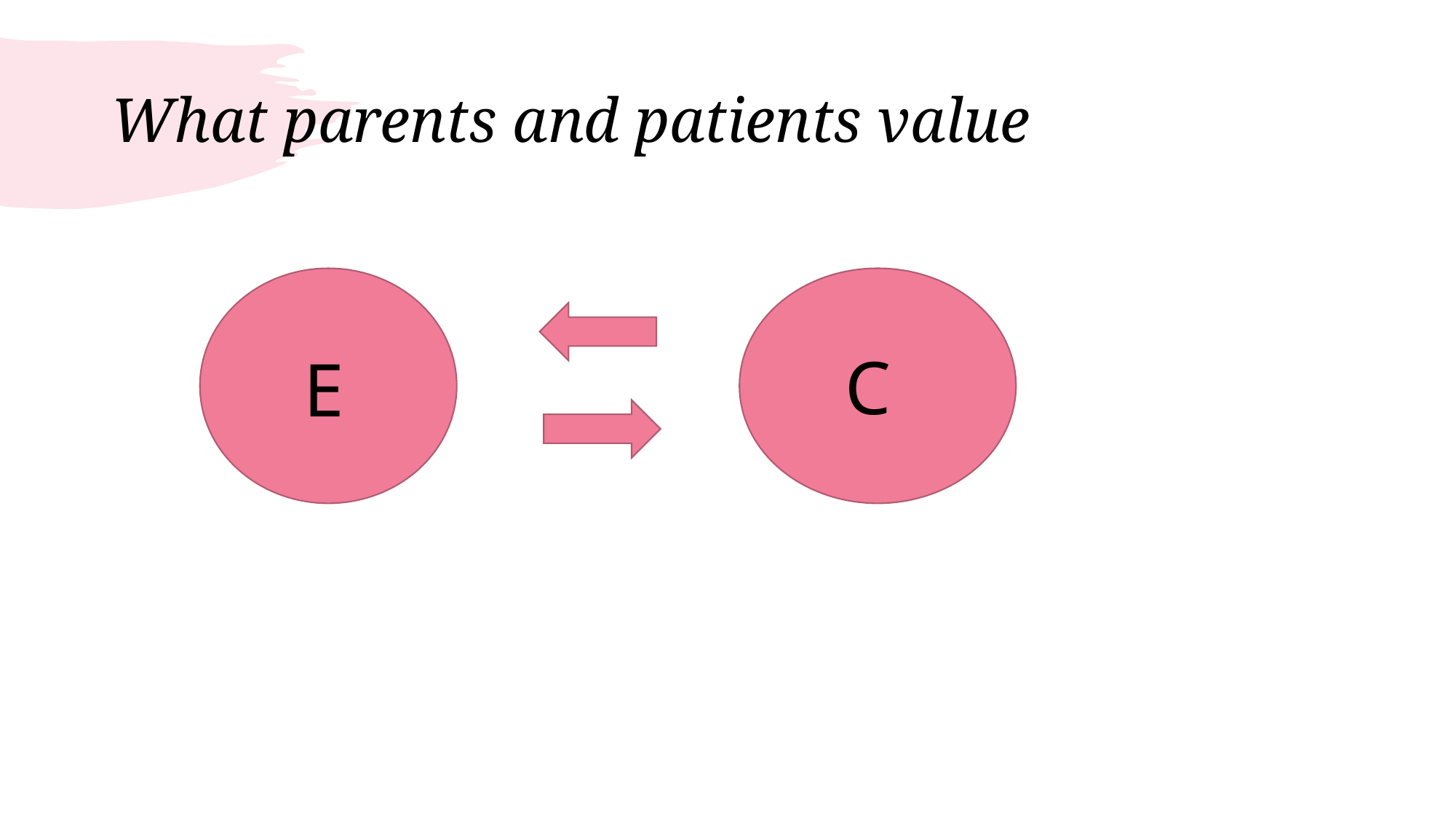

# What parents and patients value
C
E
Emotional Channel
Cognitive Channel

## Slide 9
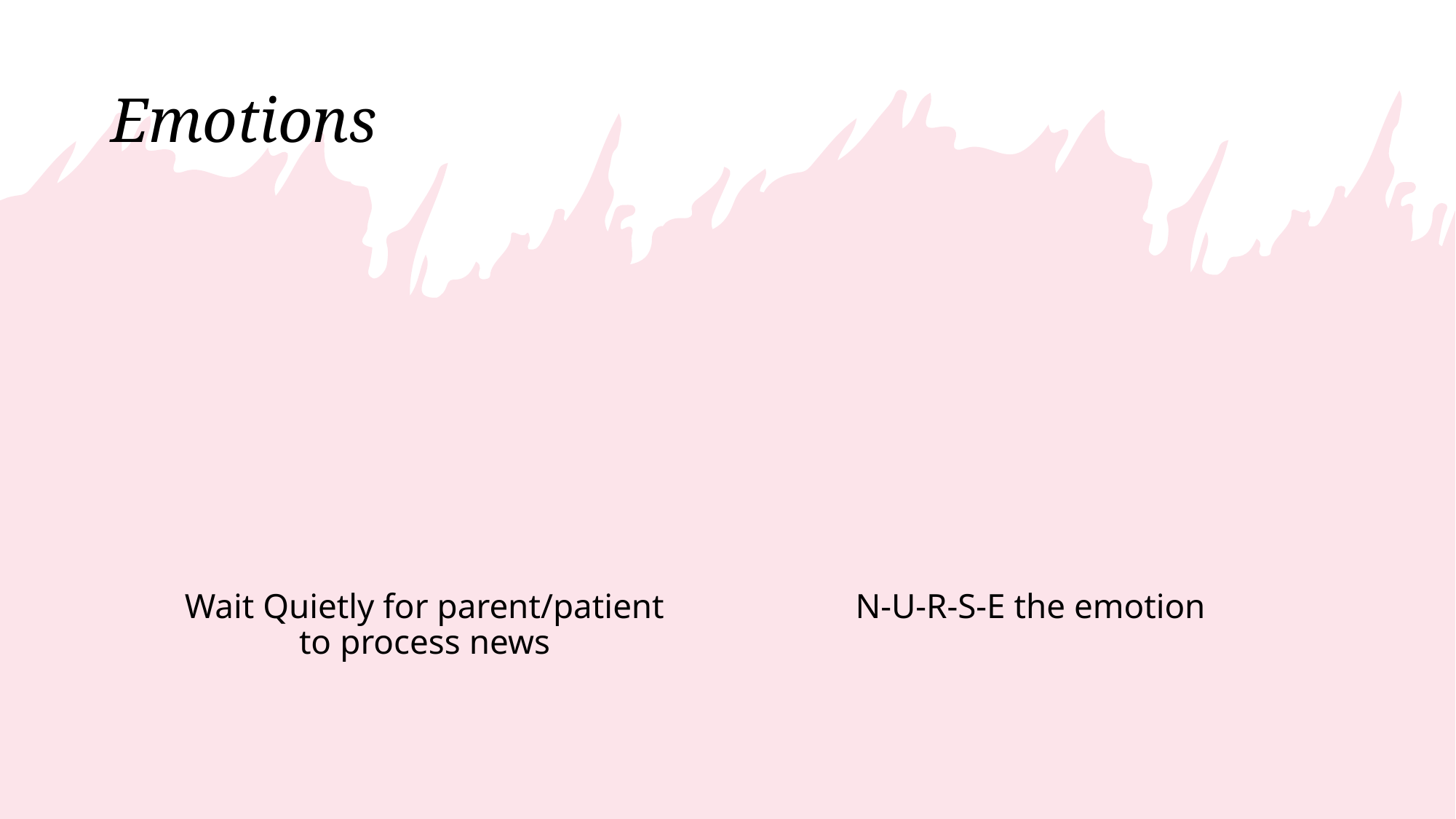

# Emotions

## Slide 10
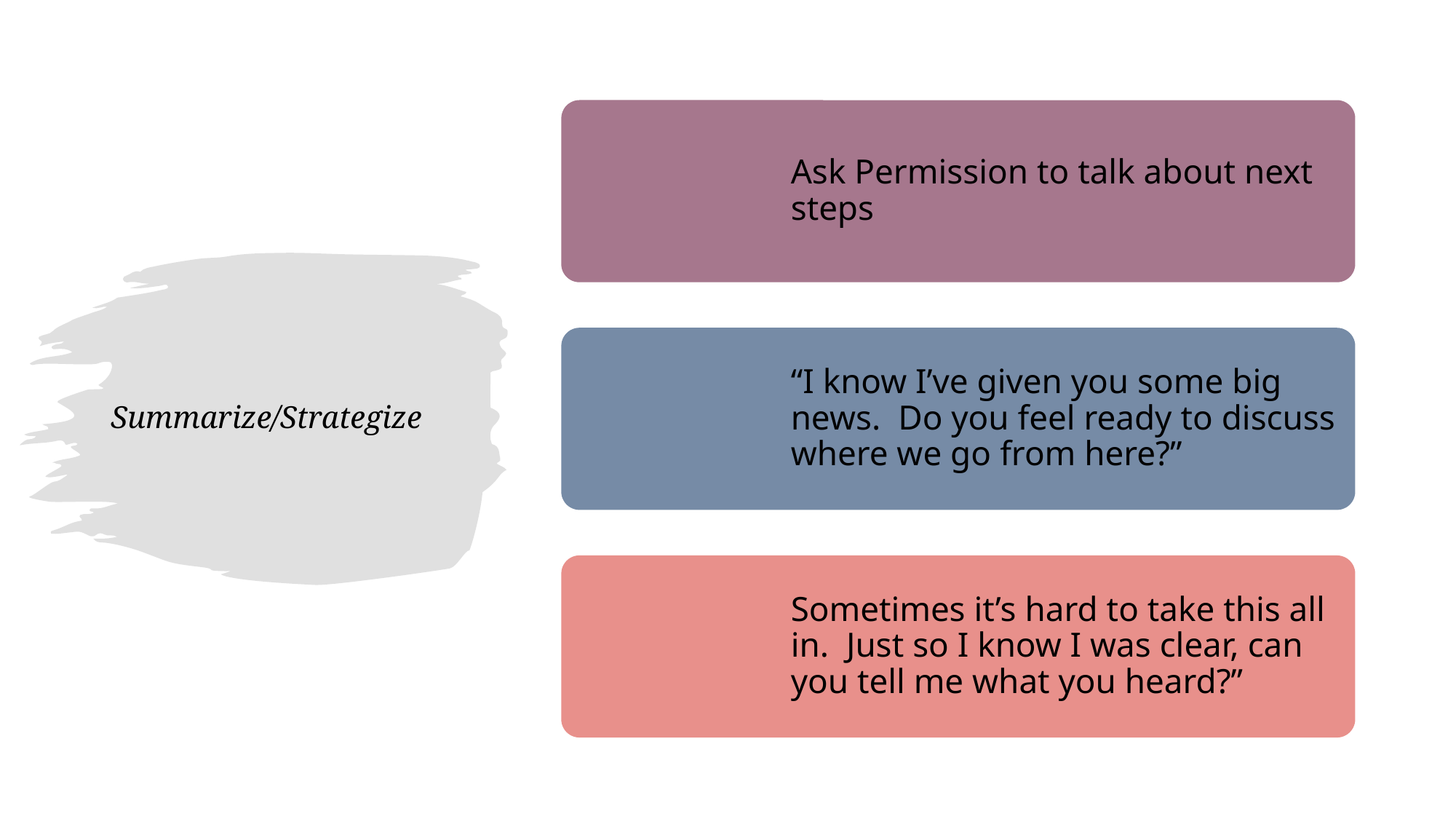

# Summarize/Strategize

## Slide 11
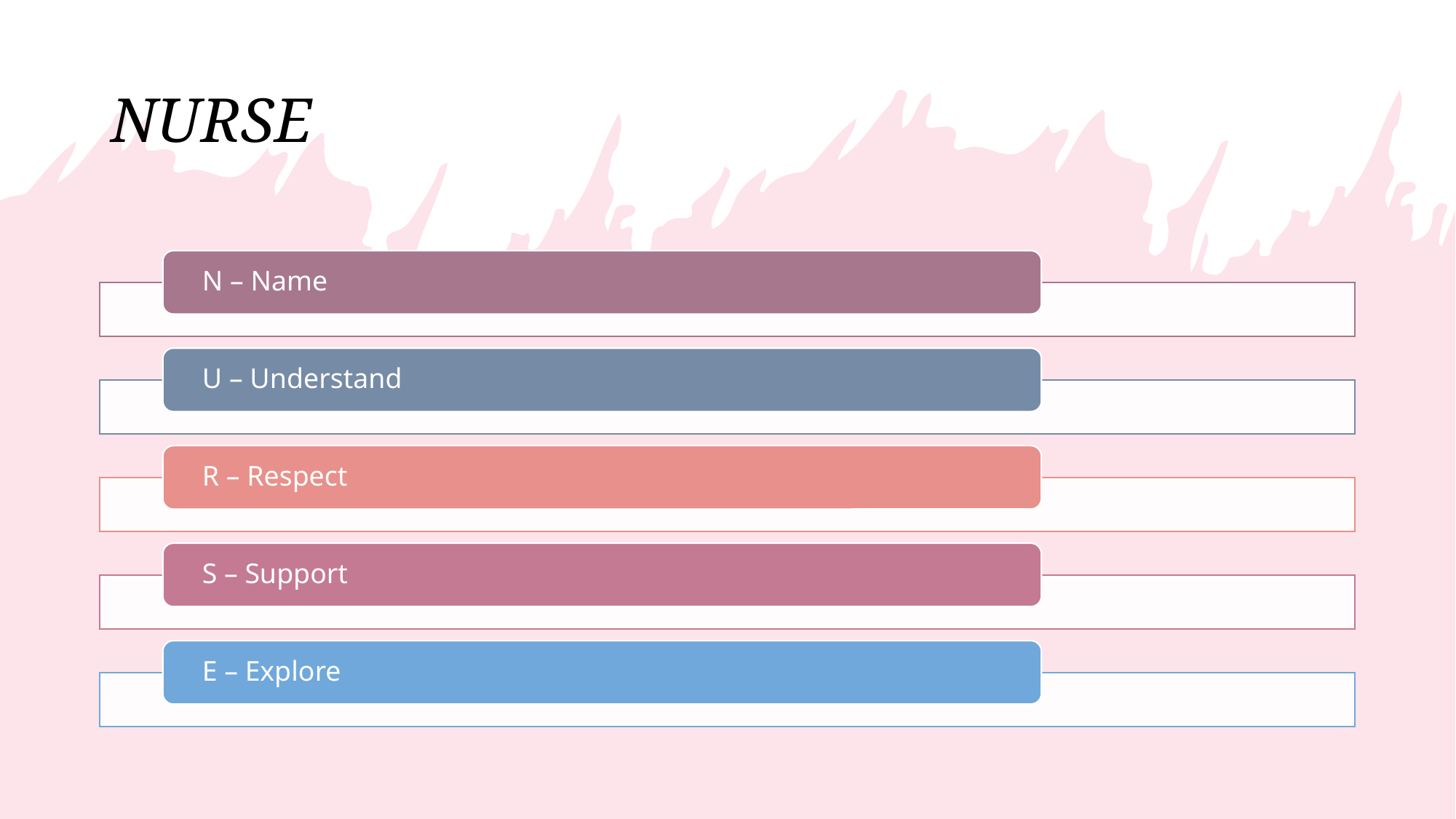

# NURSE

## Slide 12
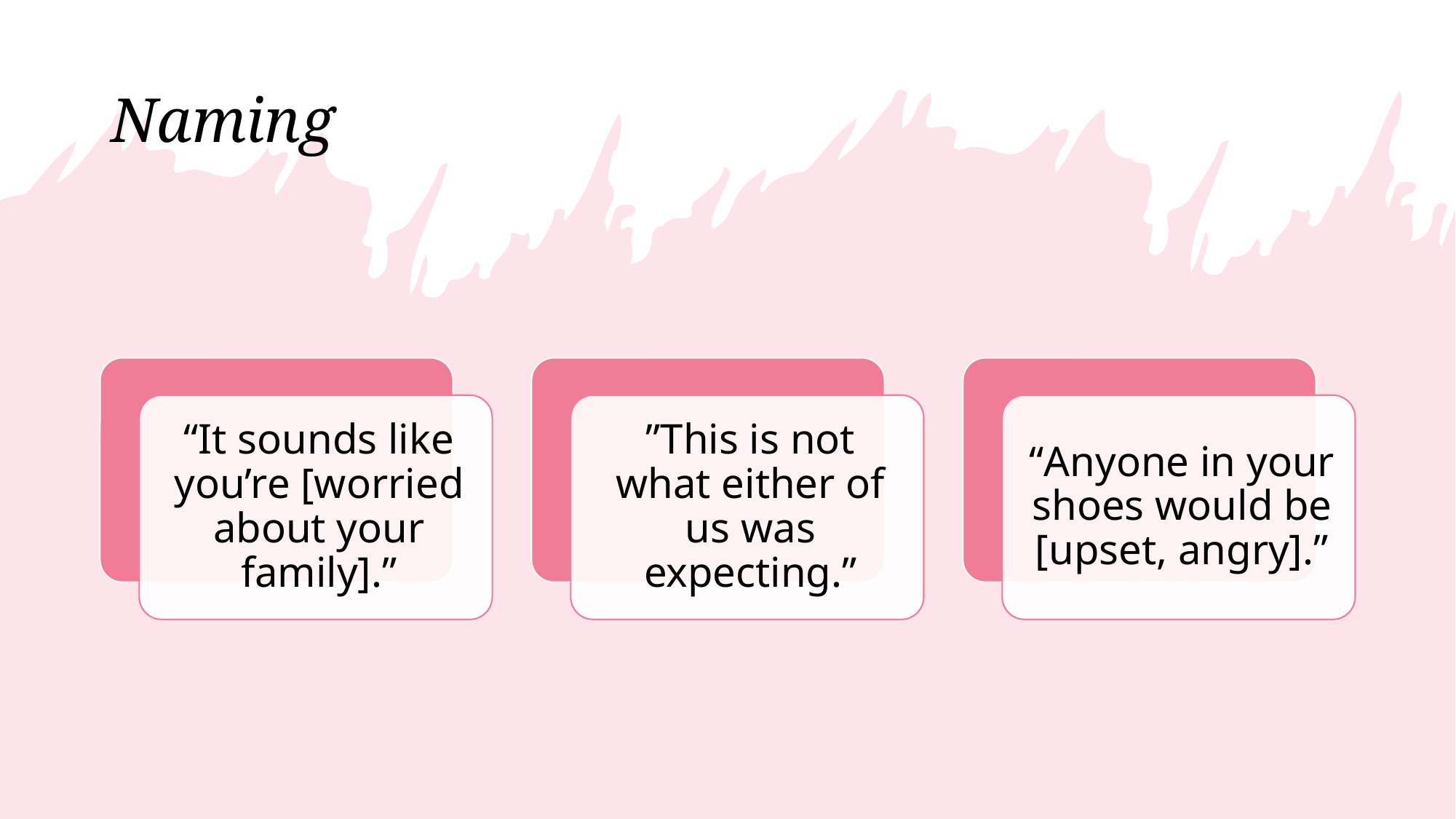

# Naming

## Slide 13
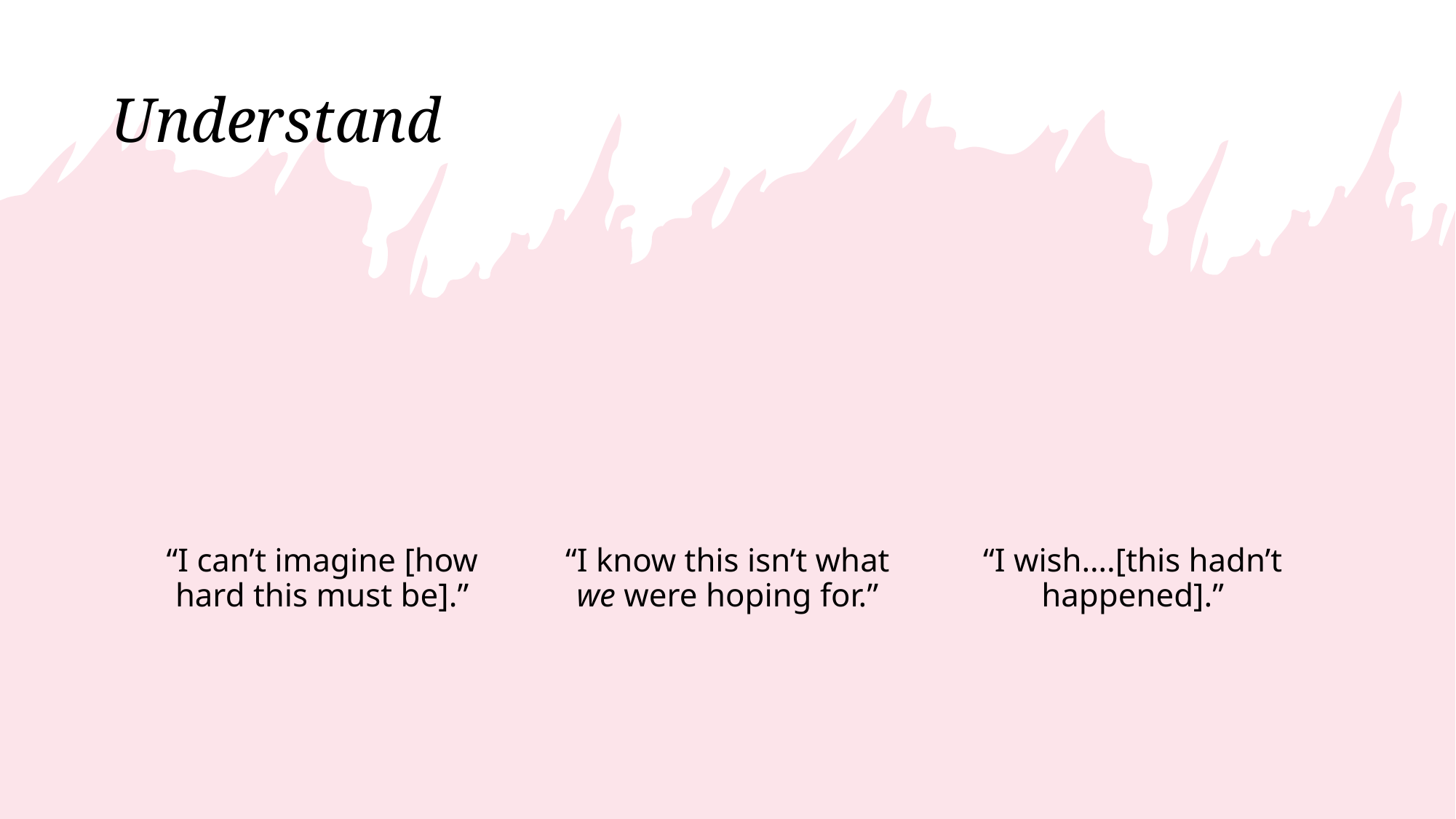

# Understand

## Slide 14
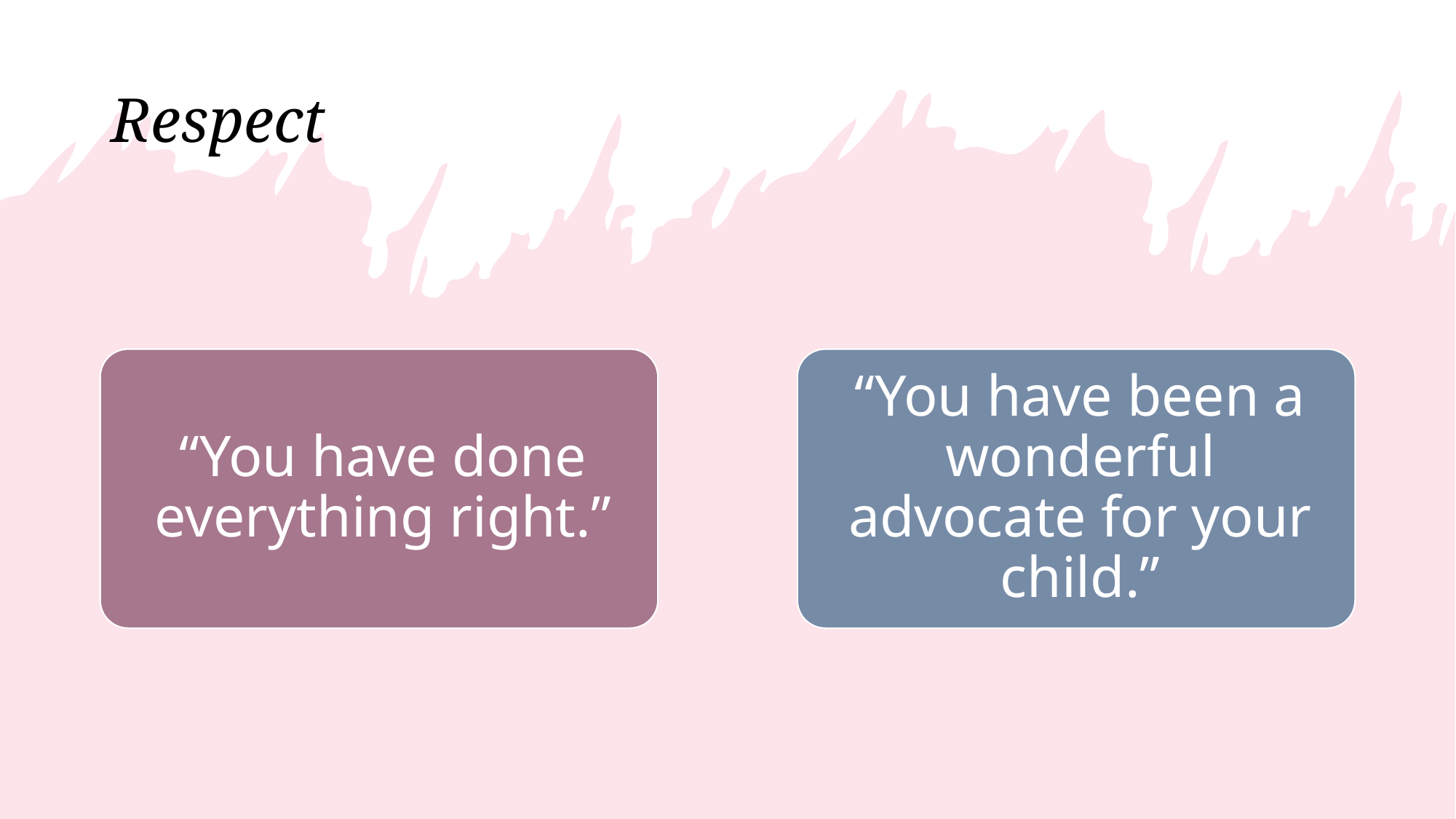

# Respect

## Slide 15
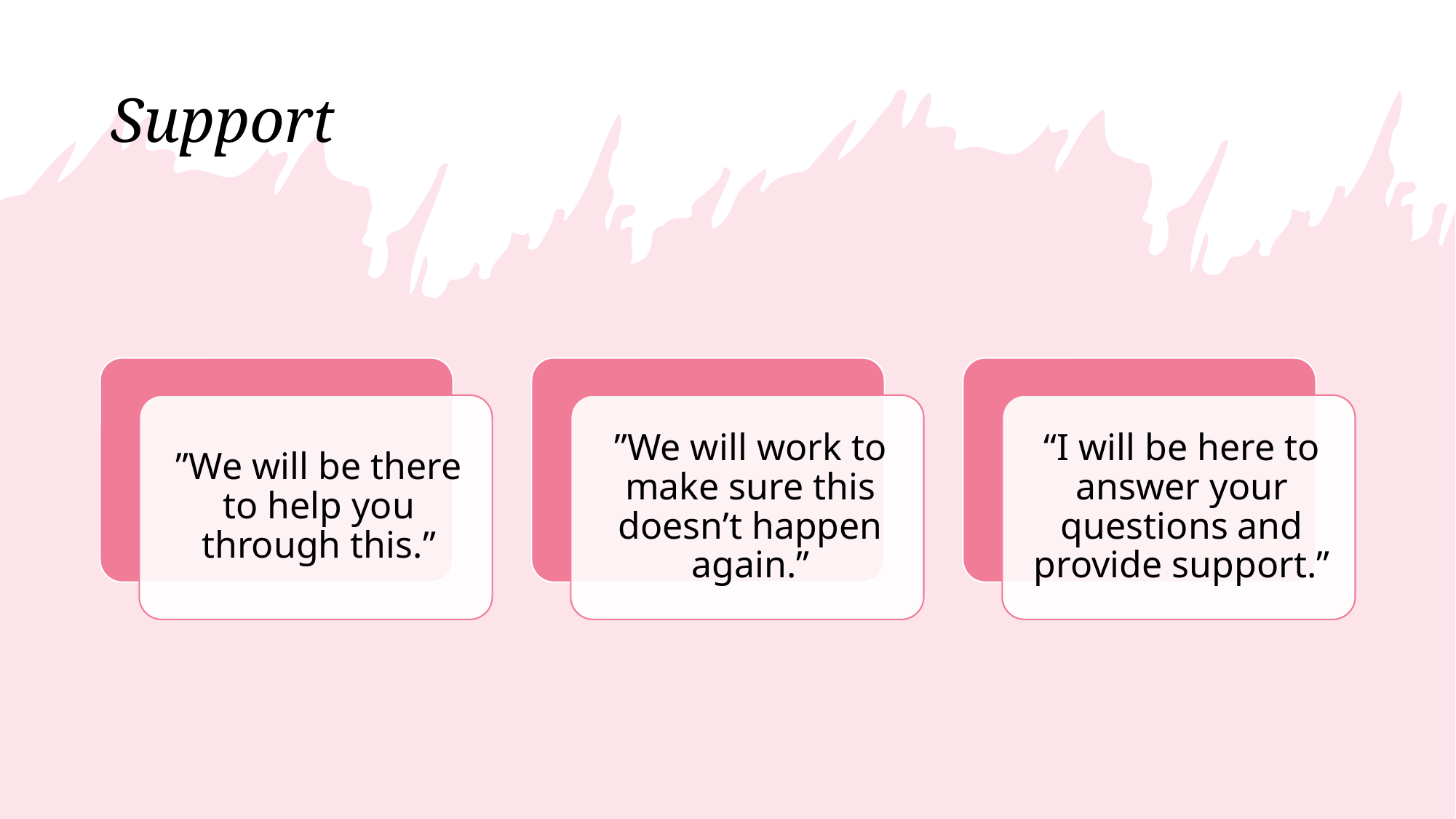

# Support

## Slide 16
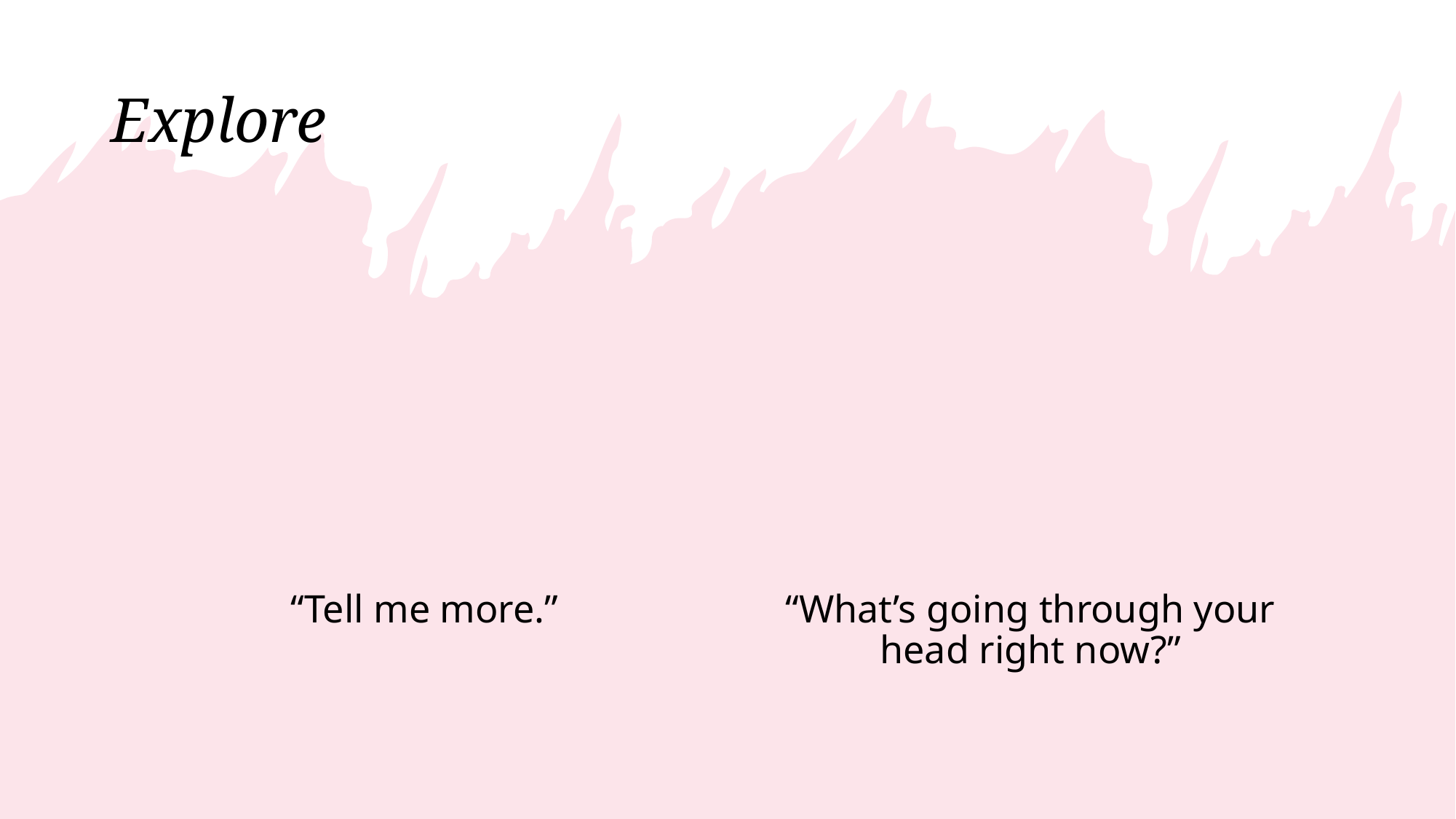

# Explore

## Slide 17
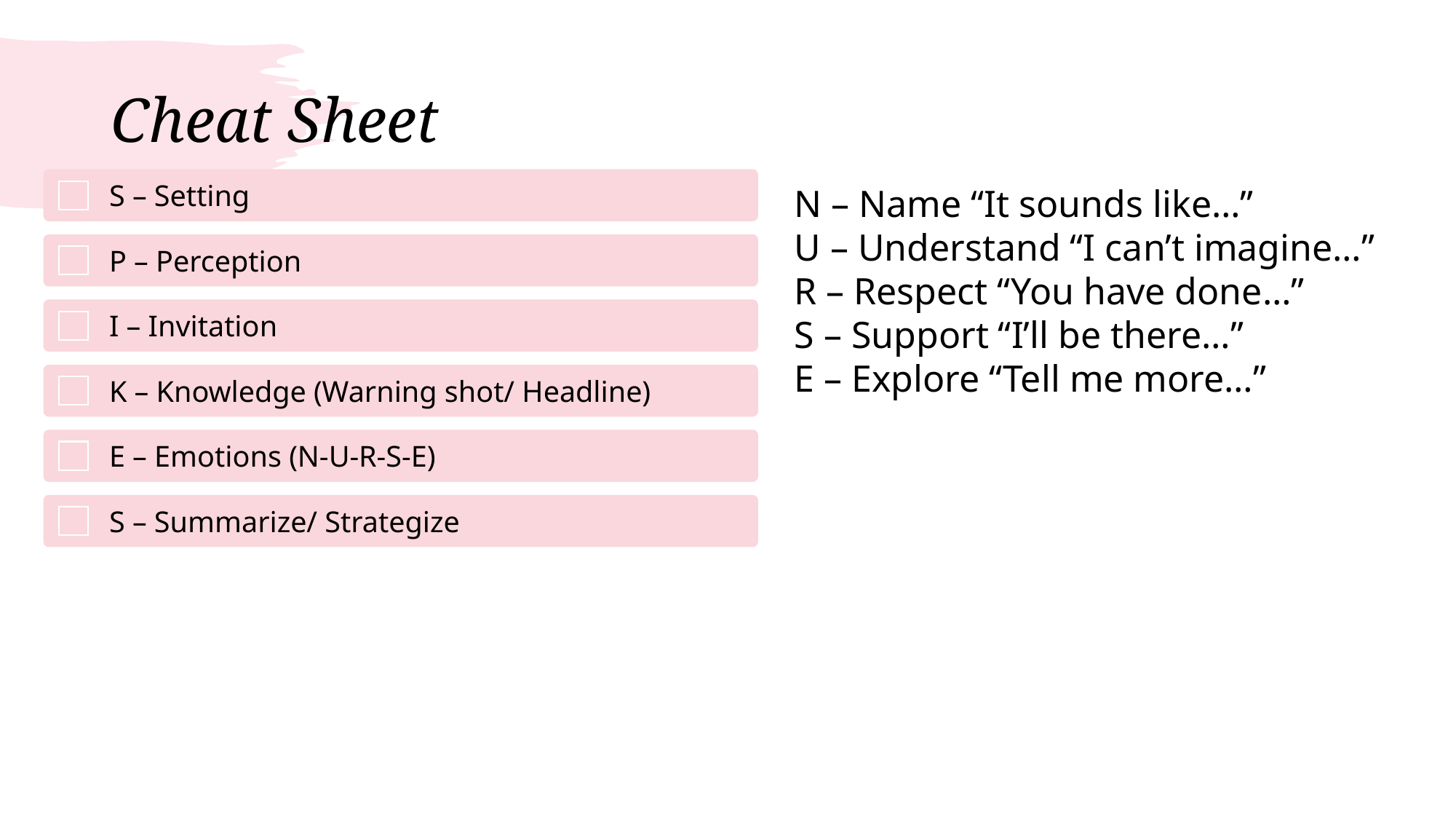

# Cheat Sheet
N – Name “It sounds like…”
U – Understand “I can’t imagine…”
R – Respect “You have done…”
S – Support “I’ll be there…”
E – Explore “Tell me more…”

## Slide 18
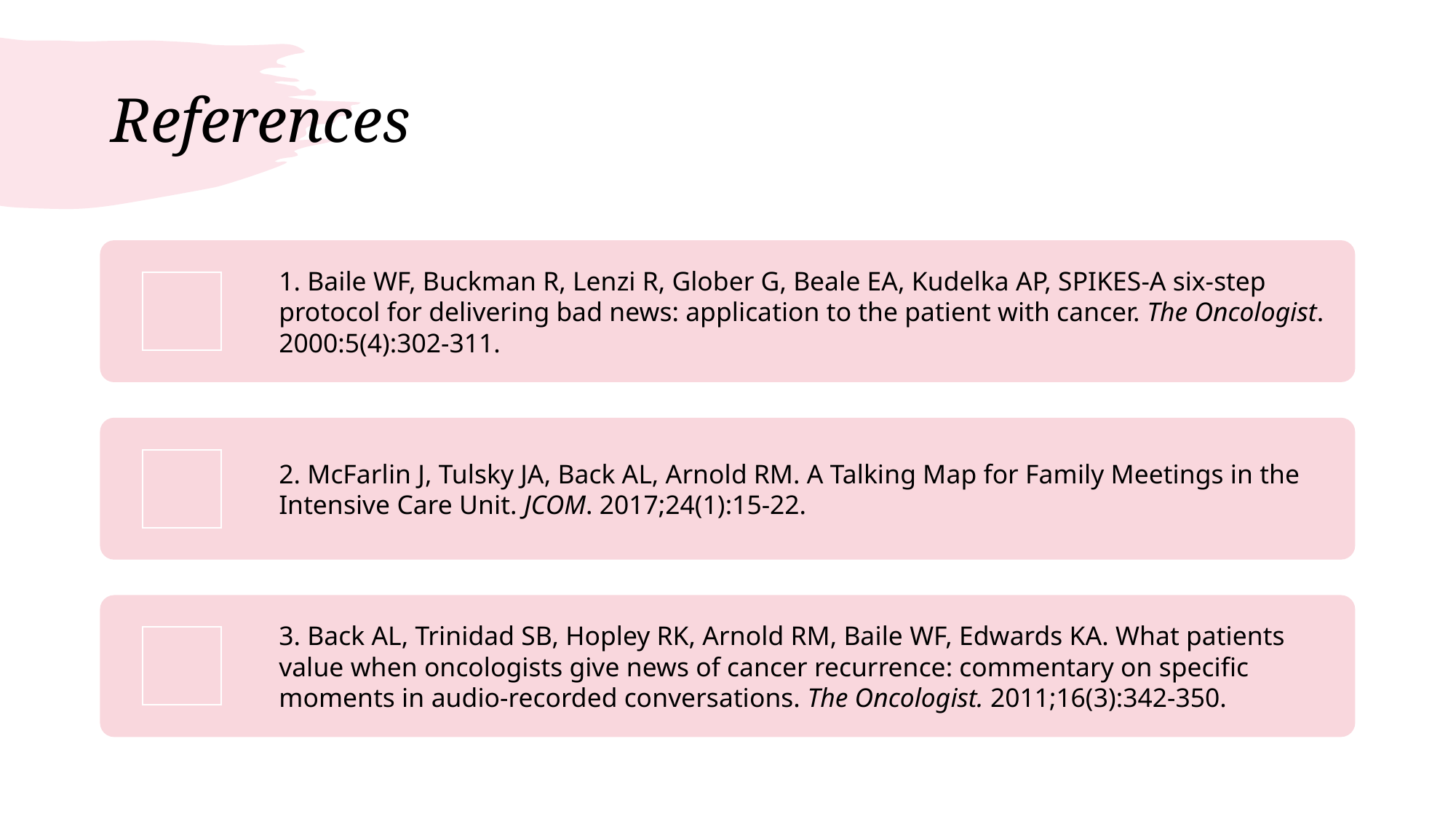

# References
