# Supplementary material for: Perioperative Difficult Conversations With Guardians of Pediatric Patients: A Simulation-Based Workshop for Anesthesiology Practitioners Using the VitalTalk Framework
Source: MedEdPORTAL. 2026 Jul 7;22:11616. doi: 10.15766/mep_2374-8265.11616 (PMC13337673; doi:10.15766/mep_2374-8265.11616)
Supplement: Supplementary file 1 — SP Handout.docxLearner Case Stems.docxSP Case for Pretest.docxSlide Deck Didactic.pptxDeliberate Practice 1 Scenario.docxDeliberate Practice 2 Scenario.docxChecklist.docxSP Case for Posttest.docxSP Case for Delayed Posttest.docxPost Course Survey.docx [file mep_2374-8265.11616-s001.zip › E. Deliberate Practice 1 Scenario.docx]

Appendix E: Deliberate Practice #1 *MedEdPORTAL* Standardized Patient Case Development Tool

This appendix contains detailed case information for the facilitator for Deliberate Practice case #1.

Date: December 9^th^, 2024

Primary Case Author: Heather Ballard MD, MS

Secondary Case Author: Mitchell Phillips MD

Standardized Patient Educator: Mitchell Phillips MD

Name of Case: Perioperative Difficult Conversations: A Simulated Patient Case Workshop for Anesthesiology Practitioners

Name of Educational and/or Assessment Activity: Adverse event- multiple intravenous catheter insertion attempts

Parent Name: Arya/Arun Patel (Child: Arjun Patel)

Chief Complaint: Seeking information after infant has required multiple intravenous catheter insertion attempts for sedation

Most Likely Diagnosis and Differential With Rationale From History and/or Physical Exam: Not applicable

Challenge Question(s):

Why did it take so many attempts to place the IV?

Couldn’t someone more experienced have done it?

Is this bruise going to cause any long-term issues?

How can we prevent this from happening again in the future?

Can you ensure my child wasn’t in too much pain?

What are the next steps in his care?

Domains: Check all that apply

X Professionalism

X Communication and Interpersonal Skills

Medical History

Physical Exam

Shared Decision-Making

X Patient Education

Clinical Reasoning

Documentation

Handoff

Presentation

Other:

Type and Level of Learner: Anesthesiology practitioners: Attending Anesthesiologists and Certified Registered Nurse Anesthetists, Anesthesiology trainees

Case Objectives: Please list specific objectives for each of the domains you have checked above:

1. Apply NURSE (naming, understanding, respecting, supporting, exploring) framework to respond to SP’s emotions with empathy and professionalism
2. Apply SPIKES (setting, perception, invitation, knowledge, emotion, summary/next steps) framework to communicate with SP about child’s multiple IV insertion attempts
3. Demonstrate SP’s understanding of child’s adverse event (need for multiple IV attempts) through education surrounding medical details of adverse event

| SETTING: outpatient, in patient, ED, home, nursing home, rehab, group, etc. | Parent of child who required multiple IV attempts for sedation to undergo magnetic resonance imaging of brain. SP is in the post anesthesia recovery unit. |
| --- | --- |
| PATIENT PROFILE: Information about the “patient” that helps select an SP and helps the learner get an understanding of them as a person. SP will know more information about the patient than learner will ever ask but allows SP to portray a fully developed patient personality. If none of the items below are particulars for the case, please write “all may be used.” | |
| Age range | 20-30 years old |
| Religious/spiritual background | All may be used |
| Sex (e.g., male, female, intersex, transwoman, transman) | All may be used |
| Sexual orientation (e.g., heterosexual, lesbian, gay, bisexual, pansexual, queer, asexual) | All may be used |
| Gender expression (e.g., man, woman, genderqueer) | All may be used |
| Race and ethnicity | All may be used |
| Physical description (e.g., BMI, height range) | All may be used |
| Physical limitations | none |
| Patient appearance (e.g., disheveled, hospital gown, business casual, casual) | Casual clothes, well kempt |
| Moulage + location (e.g., none, bruises, scars, body piercing, tattoos) | none |
| Affect (e.g., pleasant, cooperative) | Waiting for an anesthesia practitioner to talk to them in recovery room. High anxiety over possible diagnoses associated with need for MRI. Angry about the multiple IV attempts. |
| Family group (e.g., who is family, who they live with) | Lives in rural town with partner and one other child |
| Education | High school |
| Level of health literacy | Medium |
| Employment, if any - present and past, noting any current stresses | Stay at home (mom/dad) |
| Home/homeless - type of dwelling, number of stories, owned or rented | Home in rural area |
| Financial situation - any current stresses | financial stresses- far commute for imaging, worried about childcare for daughter at home |
| Insurance status (e.g., un/under/insured, public/private, HMO/PPO) | Public aid |
| Habits (i.e., diet, exercise, caffeine, smoking, alcohol, drugs) | None |
| Activities (i.e., hobbies, sports, clubs, friends) | All may be used |
| Typical day - what is the usual daily routine | Stays at home caring for child |

| CASE INFORMATION | |
| --- | --- |
| Chief Concern: What the patient will say when greeted by the student. The patient’s primary reason for seeking medical care often stated in their own words. | Don’t wait for the practitioner to introduce themselves. Ask, “What happened to my child!? Why are there so many bruises?”  . |
| Additional Concerns: Other, if any, concerns the patient has today (i.e., symptoms, requests, expectations, etc.) that will become part of set agenda. | What did the MRI show? Is my baby ok? Are these bruises going to heal? How will you stop this from happening again? |
| THE PATIENT’S STORY: The SP will be asked to tell their symptom story and the personal and emotion impact for each of their concerns. You will want to write this in the patient’s voice. The symptom story should be able to answer this question: “Tell me more about [chief concern/additional concern], starting at the beginning and bringing me up to now.”  The personal context should be able to answer questions concerning the broader personal/psychosocial context of symptoms, especially the patient’s beliefs/attributions.  The emotional context should be able to ask how are you doing with this, how does this make you feel, how has this affected you emotionally? IMPACT: How has this affected your life? How has this been for your family? | I am standing, pacing around the room. Someone better come talk to me soon. I am so upset over the appearance of my child. What is up with all these bruises? We had to drive so far to come to this hospital and I am so worried about my little guy.  The anesthesia practitioner comes to answer my questions, but I’m too distressed to hear the answers. Did I do the right thing by taking Arjun to this hospital? I’m upset by how he looks, but most of all I’m upset that my baby might be sick. I wonder if this will happen again. Will he heal from all the pokes? |
| HISTORY OF PRESENT ILLNESS: Although some of the HPI will be given in the patient’s symptom story, the learners will expand the story during the direct question section. Below, describes the detailed history, usually about the chief concern, which the student must develop to make a useful assessment of the problem: | |
| Onset (when; gradual or sudden) | Not applicable |
| Setting (what was going on or where was patient when symptoms first noticed?) | SP’s child experienced multiple attempts at IV catheter insertion |
| Duration (how long) | SP has been in the waiting room since the procedure started an hour ago. |
| Time relationships (frequency, constant or intermittent) | Not applicable. |
| Location | Not applicable |
| Radiation | Not applicable |
| Quality | Not applicable |
| Amount | Not applicable |
| Aggravated by what | Not applicable |
| Relieved by what | Not applicable |
| Associated with what | Not applicable |
| Attitude (what does the patient think is the problem, and how do they feel about it) | The SP has anxiety about their child’s diagnosis and is angry because there are bruises from all the IV insertion attempts. They display an anxious and then angry tone when the practitioner tries to explain what happened. They are worried about their son’s potential diagnosis and what will happen next.  . |
| Overall course | The SP’s son will completely recover from the bruises. The son’s diagnosis remains unknown |
| REVIEW OF SYSTEMS: Significant positives and negatives | |
| Constitutional - not applicable | Genito-urinary - not applicable |
| HEENT – not applicable | Musculoskeletal - not applicable |
| Cardiovascular – not applicable | Skin/breast - not applicable |
| Respiratory - not applicable | Neurological - not applicable |
| Gastroenterology - not applicable | Psychiatric - not applicable |
| Past medical history |  |
| Medication allergies (name and reaction) | not applicable |
| Environmental allergies (name and reaction) | not applicable |
| Illnesses | not applicable |
| Vaccinations | not applicable |
| Surgeries | not applicable |
| Accidents/injuries/trauma | not applicable |
| Hospitalization | not applicable |
|  | |
| Inclusive sexual and reproductive history | |
| Sexual practices  Sexual partners  Protection: Use of safer sex practices  Use of birth control if appropriate  Risk of intimate partner violence | not applicable |
| OB/GYN history | Age of onset of menses: not applicable  Age of menopause: not applicable  Number of pregnancies: not applicable  Number of live births: not applicable  Number of miscarriages: 0  Number of abortions: 0 |
| Medications | not applicable |
| Immunizations not applicable | X Tetanus  X Flu  X Hepatitis  X Pneumovax  X HPV  X COVID |
| Tobacco products: not applicable   - Cigarettes - Cigar - Pipe - Chew - E-cigarettes | X Never   - Past - year started/year quit - Current   - Quantity   - # of years |
| Alcohol not applicable   - Beer - Wine - Liquor - Other | X Never   - Past - year started/year quit - Current   - Quantity   - # of years |
| Drugs not applicable   - Weed - Cocaine - Heroin - Meth - IV - Inhalants - Other | X Never   - Past - year started/year quit - Current   - Quantity   - # of years |
| Diet (describe) | not applicable |
| Exercise (describe) | Not applicable |
| List any other important social history or information important to this case | Not applicable |
| Family history | not applicable |
| Mother, father, siblings, grandparents, and other significant findings | not applicable |
|  |  |
| Physical Exam – Not applicable | |
| PHYSICAL EXAM FINDINGS |  |
| 1. Written in layperson’s terms | Not applicable |
| 1. General appearance - affect, appearance, position of patient at opening (i.e., sitting, lying down, holding abdomen, etc.) | Not applicable |
| 1. Vital signs | Not applicable |
| 1. Specific findings and affect | Not applicable |
| 1. Response to certain physical movements | Not applicable |
|  |  |
| DIAGNOSIS AND DIFFERENTIAL |  |
| Diagnosis with support from positive and negative history and PE findings | Not applicable |
| Differential with support from positive and negative history and PE findings | Not applicable |
|  |  |
| MANAGEMENT OR DIAGNOSTIC PLAN | Anesthesia practitioner must inform SP that their son required multiple intravenous catheter insertion attempts under anesthesia |
|  |  |
| PROFESSIONALISM ISSUES OR CHALLENGES | Adverse event regarding multiple IV insertion attempts; Breaking bad news |
