# Supplementary material for: Perioperative Difficult Conversations With Guardians of Pediatric Patients: A Simulation-Based Workshop for Anesthesiology Practitioners Using the VitalTalk Framework
Source: MedEdPORTAL. 2026 Jul 7;22:11616. doi: 10.15766/mep_2374-8265.11616 (PMC13337673; doi:10.15766/mep_2374-8265.11616)
Supplement: Supplementary file 1 — SP Handout.docxLearner Case Stems.docxSP Case for Pretest.docxSlide Deck Didactic.pptxDeliberate Practice 1 Scenario.docxDeliberate Practice 2 Scenario.docxChecklist.docxSP Case for Posttest.docxSP Case for Delayed Posttest.docxPost Course Survey.docx [file mep_2374-8265.11616-s001.zip › F. Deliberate Practice 2 Scenario.docx]

Appendix F: Deliberate Practice #2 *MedEdPORTAL* Standardized Patient Case Development Tool

This appendix contains detailed case information for the facilitator for Deliberate Practice case #2.

Date: December 9^th^, 2024

Primary Case Author: Heather Ballard MD, MS

Secondary Case Author: Mitchell Phillips MD

Standardized Patient Educator: Mitchell Phillips MD

Name of Case: Perioperative Difficult Conversations: A Simulated Patient Case Workshop for Anesthesiology Practitioners

Name of Educational and/or Assessment Activity: Adverse event- failed caudal nerve block

Parent Name: Cecilia/Cecil Rudd (Child: Thomas Rudd)

Chief Complaint: Seeking information because infant has uncontrolled pain after hypospadias repair surgery

Most Likely Diagnosis and Differential with Rationale from History and/or Physical Exam: Not applicable

Challenge Question(s):

Why didn’t the caudal block work?

Couldn’t someone more experienced have done it?

Is it safe to give him more pain medication?

How can we ensure he won’t be in pain when we take him home?

What are the risks of the medications he’s receiving?

What are the next steps in his care?

Domains: Check all that apply

X Professionalism

X Communication and Interpersonal Skills

Medical History

Physical Exam

Shared Decision-Making

X Patient Education

Clinical Reasoning

Documentation

Handoff

Presentation

Other:

Type and Level of Learner: Anesthesiology practitioners: Attending Anesthesiologists and Certified Registered Nurse Anesthetists, Anesthesiology trainees

Case Objectives: Please list specific objectives for each of the domains you have checked above:

1. Apply NURSE (naming, understanding, respecting, supporting, exploring) framework to respond to SP’s emotions with empathy and professionalism
2. Apply SPIKES (setting, perception, invitation, knowledge, emotion, summary/next steps) framework to communicate with SP about child’s multiple IV insertion attempts
3. Demonstrate SP’s understanding of child’s adverse event (failed caudal nerve block) through education surrounding medical details of adverse event

| SETTING: outpatient, in patient, ED, home, nursing home, rehab, group, etc. | Parent of infant who is in uncontrolled pain after hypospadias repair. SP is in the post anesthesia recovery unit. |
| --- | --- |
| PATIENT PROFILE: Information about the “patient” that helps select an SP and helps the learner get an understanding of them as a person. SP will know more information about the patient than learner will ever ask but allows SP to portray a fully developed patient personality. If none of the items below are particulars for the case, please write “all may be used.” | |
| Age range | 30-40 years old |
| Religious/spiritual background | All may be used |
| Sex (e.g., male, female, intersex, transwoman, transman) | All may be used |
| Sexual orientation (e.g., heterosexual, lesbian, gay, bisexual, pansexual, queer, asexual) | All may be used |
| Gender expression (e.g., man, woman, genderqueer) | All may be used |
| Race and ethnicity | All may be used |
| Physical description (e.g., BMI, height range) | All may be used |
| Physical limitations | none |
| Patient appearance (e.g., disheveled, hospital gown, business casual, casual) | Casual clothes, well kempt |
| Moulage + location (e.g., none, bruises, scars, body piercing, tattoos) | none |
| Affect (e.g., pleasant, cooperative) | SP appears anxious and concerned, with visible signs of distress. |
| Family group (e.g., who is family, who they live with) | Lives in suburban town with partner and three children |
| Education | Some college |
| Level of health literacy | Medium |
| Employment, if any - present and past, noting any current stresses | Stay at home (mom/dad) |
| Home/homeless - type of dwelling, number of stories, owned or rented | Home in suburbs |
| Financial situation - any current stresses | Worried about childcare for children at home |
| Insurance status (e.g., un/under/insured, public/private, HMO/PPO) | HMO/PPO |
| Habits (i.e., diet, exercise, caffeine, smoking, alcohol, drugs) | None |
| Activities (i.e., hobbies, sports, clubs, friends) | All may be used |
| Typical day - what is the usual daily routine | Stays at home caring for child |

| CASE INFORMATION | |
| --- | --- |
| Chief Concern: What the patient will say when greeted by the student. The patient’s primary reason for seeking medical care often stated in their own words. | The SP greets the practitioner with a polite but tense "Hello" or "Hi," indicating their eagerness to address their concerns. Are you going to explain why my baby is in pain and what you are going to do about it? |
| Additional Concerns: Other, if any, concerns the patient has today (i.e., symptoms, requests, expectations, etc.) that will become part of set agenda. | Why didn’t the caudal block work?  Couldn’t someone more experienced have done it?  Is it safe to give him more pain medication?  How can we ensure he won’t be in pain when we take him home?  What are the risks of the medications he’s receiving?  What are the next steps in his care? |
| THE PATIENT’S STORY: The SP will be asked to tell their symptom story and the personal and emotion impact for each of their concerns. You will want to write this in the patient’s voice. The symptom story should be able to answer this question: “Tell me more about [chief concern/additional concern], starting at the beginning and bringing me up to now.”  The personal context should be able to answer questions concerning the broader personal/psychosocial context of symptoms, especially the patient’s beliefs/attributions.  The emotional context should be able to ask how are you doing with this, how does this make you feel, how has this affected you emotionally? IMPACT: How has this affected your life? How has this been for your family? | I am with my son in the recovery room after he had hypospadias repair surgery. The anesthesiologist told me he wouldn’t be in any pain if we did this caudal nerve block, but he is screaming his head off. I was hesitant to say yes to the nerve block because it’s so close to his spinal cord. I was so afraid he would get hurt and now feel foolish for saying yes. I want to know how we can keep Tommy comfortable and if he’s going to be okay. |
| HISTORY OF PRESENT ILLNESS: Although some of the HPI will be given in the patient’s symptom story, the learners will expand the story during the direct question section. Below, describes the detailed history, usually about the chief concern, which the student must develop to make a useful assessment of the problem: | |
| Onset (when; gradual or sudden) | Not applicable |
| Setting (what was going on or where was patient when symptoms first noticed?) | SP’s child is having uncontrolled pain in the recovery room after hypospadias surgery. |
| Duration (how long) | SP has been in the recovery room with her son for twenty minutes |
| Time relationships (frequency, constant or intermittent) | Not applicable. |
| Location | Not applicable |
| Radiation | Not applicable |
| Quality | Not applicable |
| Amount | Not applicable |
| Aggravated by what | Not applicable |
| Relieved by what | Not applicable |
| Associated with what | Not applicable |
| Attitude (what does the patient think is the problem, and how do they feel about it) | Now that your child’s surgery is done, the SP is surprised to see your child in pain despite the caudal block. They are upset because their child is in pain and that the medical team put their child at unnecessary risk without any benefit.  . |
| Overall course | The SP’s son will heal from surgery without sequelae |
| REVIEW OF SYSTEMS: Significant positives and negatives | |
| Constitutional -not applicable | Genito-urinary - not applicable |
| HEENT – not applicable | Musculoskeletal - not applicable |
| Cardiovascular – not applicable | Skin/breast - not applicable |
| Respiratory - not applicable | Neurological - not applicable |
| Gastroenterology - not applicable | Psychiatric - not applicable |
| Past medical history |  |
| Medication allergies (name and reaction) | not applicable |
| Environmental allergies (name and reaction) | not applicable |
| Illnesses | not applicable |
| Vaccinations | not applicable |
| Surgeries | not applicable |
| Accidents/injuries/trauma | not applicable |
| Hospitalization | not applicable |
|  | |
| Inclusive sexual and reproductive history | |
| Sexual practices  Sexual partners  Protection: Use of safer sex practices  Use of birth control if appropriate  Risk of intimate partner violence | not applicable |
| OB/GYN history | Age of onset of menses: not applicable  Age of menopause: not applicable  Number of pregnancies: not applicable  Number of live births: not applicable  Number of miscarriages: not applicable  Number of abortions: not applicable |
| Medications | None |
| Immunizations not applicable | X Tetanus  X Flu  X Hepatitis  X Pneumovax  X HPV  X COVID |
| Tobacco products: not applicable   - Cigarettes - Cigar - Pipe - Chew - E-cigarettes | X Never   - Past - year started/year quit - Current   - Quantity   - # of years |
| Alcohol not applicable   - Beer - Wine - Liquor - Other | X Never   - Past - year started/year quit - Current   - Quantity   - # of years |
| Drugs not applicable   - Weed - Cocaine - Heroin - Meth - IV - Inhalants - Other | X Never   - Past - year started/year quit - Current   - Quantity   - # of years |
| Diet (describe) | not applicable |
| Exercise (describe) | Not applicable |
| List any other important social history or information important to this case | Not applicable |
| Family history |  |
| Mother, father, siblings, grandparents, and other significant findings | not applicable |
|  |  |
| Physical Exam – Not applicable | |
| PHYSICAL EXAM FINDINGS |  |
| 1. Written in layperson’s terms | Not applicable |
| 1. General appearance - affect, appearance, position of patient at opening (i.e., sitting, lying down, holding abdomen, etc.) | Not applicable |
| 1. Vital signs | Not applicable |
| 1. Specific findings and affect | Not applicable |
| 1. Response to certain physical movements | Not applicable |
|  |  |
| DIAGNOSIS AND DIFFERENTIAL |  |
| Diagnosis with support from positive and negative history and PE findings | Not applicable |
| Differential with support from positive and negative history and PE findings | Not applicable |
|  |  |
| MANAGEMENT OR DIAGNOSTIC PLAN | Anesthesia practitioner must inform SP that their son’s nerve block was not effective and develop a plan for postoperative pain control |
|  |  |
| PROFESSIONALISM ISSUES OR CHALLENGES | Adverse event regarding failed nerve block; Breaking bad news |
