# Supplementary material for: Perioperative Difficult Conversations With Guardians of Pediatric Patients: A Simulation-Based Workshop for Anesthesiology Practitioners Using the VitalTalk Framework
Source: MedEdPORTAL. 2026 Jul 7;22:11616. doi: 10.15766/mep_2374-8265.11616 (PMC13337673; doi:10.15766/mep_2374-8265.11616)
Supplement: Supplementary file 1 — SP Handout.docxLearner Case Stems.docxSP Case for Pretest.docxSlide Deck Didactic.pptxDeliberate Practice 1 Scenario.docxDeliberate Practice 2 Scenario.docxChecklist.docxSP Case for Posttest.docxSP Case for Delayed Posttest.docxPost Course Survey.docx [file mep_2374-8265.11616-s001.zip › G. Checklist.docx]

Appendix G: Breaking Bad News Skill Checklist

|  | **Yes** | **No** |
| --- | --- | --- |
| **Creates initial rapport when first walking into room (e.g. introduces self/introduced by someone)** |  |  |
| **Sits down** |  |  |
| **Assumes a comfortable interpersonal distance** |  |  |
| **Assesses family’s perception or understanding of medical situation before breaking news (e.g. “tell me what you understand”)** |  |  |
| **Asks permission before giving the news (e.g. “I would like to discuss what happened”)** |  |  |
| **Gives a clear and concise “warning shot” (e.g. “I have some serious news”)** |  |  |
| **Pauses after delivering bad news** |  |  |
| **Delivers bad news within the first minute of the conversation** |  |  |
| **Delivers an empathic statement (e.g. “I know this is not what you expected to hear today”)** |  |  |
| **Suggests a plan for the next step** |  |  |
| **Ensures family understanding (e.g. “It sounds like …”)** |  |  |
| **Avoids medical jargon (uses technical language without clarifying what it means)** |  |  |
| **Gives information in small chunks (e.g. no more than 1 chunk of information before allowing family to process)** |  |  |
| **Avoids giving information while family very emotional** |  |  |
| **Avoids providing reassurances to family’s emotion (e.g. avoid saying something like “it’s ok”)** |  |  |
| **Listens attentively** |  |  |

**Breaking Bad News Skill Checklist^a^**

**^a^** This checklist was adapted from our previous work: Phillips ML, Tsao M, Davis-Sandfoss A, et al. Use of Simulation-Based Mastery Learning Curriculum to Improve Difficult Conversation Skills Among Anesthesiologists: A Pilot Study. *J Educ Perioper Med*. 2023;25(3):E710. Published 2023 Jul 1. doi:10.46374/volxxv_issue3_Phillips
