# Supplementary material for: Perioperative Difficult Conversations With Guardians of Pediatric Patients: A Simulation-Based Workshop for Anesthesiology Practitioners Using the VitalTalk Framework
Source: MedEdPORTAL. 2026 Jul 7;22:11616. doi: 10.15766/mep_2374-8265.11616 (PMC13337673; doi:10.15766/mep_2374-8265.11616)
Supplement: Supplementary file 1 — SP Handout.docxLearner Case Stems.docxSP Case for Pretest.docxSlide Deck Didactic.pptxDeliberate Practice 1 Scenario.docxDeliberate Practice 2 Scenario.docxChecklist.docxSP Case for Posttest.docxSP Case for Delayed Posttest.docxPost Course Survey.docx [file mep_2374-8265.11616-s001.zip › J. Post Course Survey.docx]

**Appendix J: Post Course Learner Survey**

**Name: __________________**

**Date: __________________**

**Post Course Survey Evaluation**

1. What is your current level of training? (Post-graduate year level OR current role if post-licensure) __________________________________________
2. If applicable, years of clinical practice since completion of training ________________
3. Have you ever had to break bad news before? CIRCLE ONE: Yes No
4. Roughly, how many times would you say you have had to break bad news to a patient or family?

NEVER <3 3-5 6-8 9-10 >10 (or just leave a number) _____

1. Have you ever had any formal training on how to break bad news?

CIRCLE ONE: Yes No

1. At what level of education did you complete formal training on how to break bad news?

CIRCLE all applicable

SRNA/Medical school Residency CRNA Attending No formal training Other

If other, please describe: _______________________________________________

1. What was your overall **confidence** with breaking bad news **BEFORE** the simulation?

Circle: 1 2 3 4 5

1. What is your overall **confidence** with breaking bad news **AFTER** the simulation?

Circle: 1 2 3 4 5

1. What is your overall satisfaction with the simulation “breaking bad news”?

Circle: 1 2 3 4 5

1. What is your overall satisfaction of the standardized parents?

Circle: 1 2 3 4 5

1. What is your overall satisfaction of the lecture?

Circle: 1 2 3 4 5

1. How could we improve the simulation “breaking bad news”? (Free response)

Thank you for participating in this simulation!
