# Supplementary material for: Perioperative Difficult Conversations With Guardians of Pediatric Patients: A Simulation-Based Workshop for Anesthesiology Practitioners Using the VitalTalk Framework
Source: MedEdPORTAL. 2026 Jul 7;22:11616. doi: 10.15766/mep_2374-8265.11616 (PMC13337673; doi:10.15766/mep_2374-8265.11616)
Supplement: Supplementary file 1 — SP Handout.docxLearner Case Stems.docxSP Case for Pretest.docxSlide Deck Didactic.pptxDeliberate Practice 1 Scenario.docxDeliberate Practice 2 Scenario.docxChecklist.docxSP Case for Posttest.docxSP Case for Delayed Posttest.docxPost Course Survey.docx [file mep_2374-8265.11616-s001.zip › C. SP Case for Pretest.docx]

Appendix C: Pre-test Encounter *MedEdPORTAL* Standardized Patient Case Development Tool

This appendix contains detailed case information for the facilitator for the Pre-test encounter.

Date: December 9^th^, 2024

Primary Case Author: Heather Ballard MD, MS

Secondary Case Author: Mitchell Phillips MD

Standardized Patient Educator: Mitchell Phillips MD

Name of Case: Perioperative Difficult Conversations: A Simulated Patient Case Workshop for Anesthesiology Practitioners

Name of Educational and/or Assessment Activity: Adverse event- unplanned intensive care unit admission

Parent Name: Calie/Shaun Wolfe (Child: Willa Wolfe)

Chief Complaint: Seeking information after daughter has unexpected admission to intensive care unit

Most Likely Diagnosis and Differential With Rationale From History and/or Physical Exam: Not applicable

Challenge Question(s):

What exactly happened to my child? How could such a mistake occur?

Is my child going to be okay?

How long will my child need to stay in the ICU?

What are you doing to ensure this doesn’t happen again?

Can I see my child now?

Who is responsible for this error?

Domains: Check all that apply

X Professionalism

X Communication and Interpersonal Skills

Medical History

Physical Exam

Shared Decision-Making

X Patient Education

Clinical Reasoning

Documentation

Handoff

Presentation

Other:

Type and Level of Learner: All Anesthesiology practitioners: Attending Anesthesiologists, Certified Registered Nurse Anesthetists, Anesthesiology trainees

Case Objectives: Please list specific objectives for each of the domains you have checked above:

1. Apply NURSE (naming, understanding, respecting, supporting, exploring) framework to respond to SP’s emotions with empathy and professionalism
2. Apply SPIKES (setting, perception, invitation, knowledge, emotion, summary/next steps) framework to communicate with SP about child’s unexpected intensive care unit admission
3. Demonstrate Simulated Parent’s understanding of child’s adverse event (intensive care unit admission) through education surrounding medical details of adverse event

| SETTING: outpatient, in patient, ED, home, nursing home, rehab, group, etc. | Parent of child who was recently admitted to the intensive care unit after surgery. Simulated Parent is in a private waiting room outside of the operating room. |
| --- | --- |
| PATIENT PROFILE: Information about the “patient” that helps select an SP and helps the learner get an understanding of them as a person. SP will know more information about the patient than learner will ever ask but allows SP to portray a fully developed patient personality. If none of the items below are particulars for the case, please write “all may be used.” | |
| Age range | 30-40 years old |
| Religious/spiritual background | All may be used |
| Sex (e.g., male, female, intersex, transwoman, transman) | All may be used |
| Sexual orientation (e.g., heterosexual, lesbian, gay, bisexual, pansexual, queer, asexual) | All may be used |
| Gender expression (e.g., man, woman, genderqueer) | All may be used |
| Race and ethnicity | All may be used |
| Physical description (e.g., BMI, height range) | All may be used |
| Physical limitations | none |
| Patient appearance (e.g., disheveled, hospital gown, business casual, casual) | Business clothes, well kempt |
| Moulage + location (e.g., none, bruises, scars, body piercing, tattoos) | none |
| Affect (e.g., pleasant, cooperative) | Waiting for their child to come back after surgery. Reading book/watching TV. Initially confused where their child is. Quickly switches to anger. |
| Family group (e.g., who is family, who they live with) | Lives in suburbs with partner and only child who is having elective surgery |
| Education | Graduate school |
| Level of health literacy | High |
| Employment, if any - present and past, noting any current stresses | Lawyer |
| Home/homeless - type of dwelling, number of stories, owned or rented | Home in suburbs |
| Financial situation - any current stresses | no financial stresses |
| Insurance status (e.g., un/under/insured, public/private, HMO/PPO) | private |
| Habits (i.e., diet, exercise, caffeine, smoking, alcohol, drugs) | None |
| Activities (i.e., hobbies, sports, clubs, friends) | All may be used |
| Typical day - what is the usual daily routine | goes to work daily, spends time with partner (if applicable) and baby when at home |

| CASE INFORMATION | |
| --- | --- |
| Chief Concern: What the patient will say when greeted by the student. The patient’s primary reason for seeking medical care often stated in their own words. | Hi. How did the surgery go? Is everything okay with my child?  The parent should initially display a hopeful and expectant demeanor, anticipating positive news about their child's surgery. Upon hearing the practitioner’s entry, they might greet them politely but with a touch of anxiety, reflecting their concern for their child's well-being. |
| Additional Concerns: Other, if any, concerns the patient has today (i.e., symptoms, requests, expectations, etc.) that will become part of set agenda. | Is their child going to recover from this procedure? How can the mistake be avoided again? Will they have to pay for the intensive care unit stay? |
| THE PATIENT’S STORY: The SP will be asked to tell their symptom story and the personal and emotion impact for each of their concerns. You will want to write this in the patient’s voice. The symptom story should be able to answer this question: “Tell me more about [chief concern/additional concern], starting at the beginning and bringing me up to now.”  The personal context should be able to answer questions concerning the broader personal/psychosocial context of symptoms, especially the patient’s beliefs/attributions.  The emotional context should be able to ask how are you doing with this, how does this make you feel, how has this affected you emotionally? IMPACT: How has this affected your life? How has this been for your family? | I am sitting in the waiting room awaiting the arrival of my daughter’s surgeon to see how she’s doing. She is my only child and the sweetest little baby. I was nervous about my baby having surgery, but we were told that the hernias needed to be fixed.  There’s a knock at the door and I am surprised to see my anesthesia practitioner and not my surgeon. I am expecting that they will tell me about the anesthesia care during the surgery and tell me that I can go to the recovery room to see my baby. Instead, they tell me that my daughter received too much medicine and needs to stay in the intensive care unit until it wears off. Initially, I’m confused because this doesn’t make sense to me. This was supposed to be routine elective surgery, and we were planning on going home today. Now, I am hopping mad that my child had a medication error and needed to go to the intensive care unit. This anesthesia practitioner better answer my questions quickly. |
| HISTORY OF PRESENT ILLNESS: Although some of the HPI will be given in the patient’s symptom story, the learners will expand the story during the direct question section. Below, describes the detailed history, usually about the chief concern, which the student must develop to make a useful assessment of the problem: | |
| Onset (when; gradual or sudden) | Not applicable |
| Setting (what was going on or where was patient when symptoms first noticed?) | SP’s child experienced an intraoperative medication error requiring an intensive care unit admission. |
| Duration (how long) | SP has been in the waiting room since the procedure started an hour ago. |
| Time relationships (frequency, constant or intermittent) | Not applicable. |
| Location | Not applicable |
| Radiation | Not applicable |
| Quality | Not applicable |
| Amount | Not applicable |
| Aggravated by what | Not applicable |
| Relieved by what | Not applicable |
| Associated with what | Not applicable |
| Attitude (what does the patient think is the problem, and how do they feel about it) | The SP has anxiety about their child needing a surgery. They display a confused and then angry tone after news of the medication error. They are worried about whether their daughter will fully recover and when the breathing tube will be removed  . |
| Overall course | The SP’s daughter will completely recover but needs to spend extra time in the intensive care unit until the muscle relaxant wears off and the breathing tube can be removed. |
| REVIEW OF SYSTEMS: Significant positives and negatives | |
| Constitutional - not applicable | Genito-urinary - not applicable |
| HEENT – not applicable | Musculoskeletal - not applicable |
| Cardiovascular –not applicable | Skin/breast -not applicable |
| Respiratory - not applicable | Neurological -not applicable |
| Gastroenterology - not applicable | Psychiatric - not applicable |
| Past medical history |  |
| Medication allergies (name and reaction) | not applicable |
| Environmental allergies (name and reaction) | not applicable |
| Illnesses | not applicable |
| Vaccinations | not applicable |
| Surgeries | not applicable |
| Accidents/injuries/trauma | not applicable |
| Hospitalization | not applicable |
|  | |
| Inclusive sexual and reproductive history | |
| Sexual practices  Sexual partners  Protection: Use of safer sex practices  Use of birth control if appropriate  Risk of intimate partner violence | not applicable |
| OB/GYN history | Age of onset of menses: not applicable  Age of menopause: not applicable  Number of pregnancies: not applicable  Number of live births: not applicable  Number of miscarriages: not applicable  Number of abortions: not applicable |
| Medications | not applicable |
| Immunizations not applicable | X Tetanus  X Flu  X Hepatitis  X Pneumovax  X HPV  X COVID |
| Tobacco products: not applicable   - Cigarettes - Cigar - Pipe - Chew - E-cigarettes | X Never   - Past - year started/year quit - Current   - Quantity   - # of years |
| Alcohol not applicable   - Beer - Wine - Liquor - Other | X Never   - Past - year started/year quit - Current   - Quantity   - # of years |
| Drugs not applicable   - Weed - Cocaine - Heroin - Meth - IV - Inhalants - Other | X Never   - Past - year started/year quit - Current   - Quantity   - # of years |
| Diet (describe) | not applicable |
| Exercise (describe) | not applicable |
| List any other important social history or information important to this case | Not applicable |
| Family history |  |
| Mother, father, siblings, grandparents, and other significant findings | not applicable |
|  |  |
| Physical Exam – Not applicable | |
| PHYSICAL EXAM FINDINGS |  |
| 1. Written in layperson’s terms | Not applicable |
| 1. General appearance - affect, appearance, position of patient at opening (i.e., sitting, lying down, holding abdomen, etc.) | Not applicable |
| 1. Vital signs | Not applicable |
| 1. Specific findings and affect | Not applicable |
| 1. Response to certain physical movements | Not applicable |
|  |  |
| DIAGNOSIS AND DIFFERENTIAL |  |
| Diagnosis with support from positive and negative history and PE findings | Not applicable |
| Differential with support from positive and negative history and PE findings | Not applicable |
|  |  |
| MANAGEMENT OR DIAGNOSTIC PLAN | Anesthesia practitioner must inform SP that their daughter was given too much muscle relaxant and needs to recover in the intensive care unit. |
|  |  |
| PROFESSIONALISM ISSUES OR CHALLENGES | Adverse event regarding unplanned intensive care unit admission; Breaking bad news |
