# Supplementary material for: Perioperative Difficult Conversations With Guardians of Pediatric Patients: A Simulation-Based Workshop for Anesthesiology Practitioners Using the VitalTalk Framework
Source: MedEdPORTAL. 2026 Jul 7;22:11616. doi: 10.15766/mep_2374-8265.11616 (PMC13337673; doi:10.15766/mep_2374-8265.11616)
Supplement: Supplementary file 1 — SP Handout.docxLearner Case Stems.docxSP Case for Pretest.docxSlide Deck Didactic.pptxDeliberate Practice 1 Scenario.docxDeliberate Practice 2 Scenario.docxChecklist.docxSP Case for Posttest.docxSP Case for Delayed Posttest.docxPost Course Survey.docx [file mep_2374-8265.11616-s001.zip › H. SP Case for Posttest.docx]

Appendix H: Post-test Encounter *MedEdPORTAL* Standardized Patient Case Development Tool

This appendix contains detailed case information for the facilitator for the Post-test encounter.

Date: December 9^th^, 2024

Primary Case Author: Heather Ballard MD, MS

Secondary Case Author: Mitchell Phillips MD

Standardized Patient Educator: Mitchell Phillips MD

Name of Case: Perioperative Difficult Conversations: A Simulated Patient Case Workshop for Anesthesiology Practitioners

Name of Educational and/or Assessment Activity: Adverse event- wrong medication administration

Parent Name: Deborah/Daniel Smith, (Child: Carson Smith)

Chief Complaint: Seeking information after son has allergic reaction from wrong antibiotic administration

Most Likely Diagnosis and Differential With Rationale From History and/or Physical Exam: Not applicable

Challenge Question(s):

How could this mistake happen when it’s clearly documented in his medical record?

Is my child going to be okay?

What are the immediate and long-term effects of this reaction?

How will you ensure this doesn’t happen again?

Who is responsible for this error?

Domains: Check all that apply

X Professionalism

X Communication and Interpersonal Skills

Medical History

Physical Exam

Shared Decision-Making

X Patient Education

Clinical Reasoning

Documentation

Handoff

Presentation

Other:

Type and Level of Learner: Anesthesiology practitioners: Attending Anesthesiologists and Certified Registered Nurse Anesthetists

Case Objectives: Please list specific objectives for each of the domains you have checked above:

1. Apply NURSE (naming, understanding, respecting, supporting, exploring) framework to respond to SP’s emotions with empathy and professionalism
2. Apply SPIKES (setting, perception, invitation, knowledge, emotion, summary/next steps) framework to communicate with SP about child’s adverse event (allergic reaction).
3. Demonstrate SP’s understanding of child’s adverse event (allergic reaction) through education surrounding medical details of adverse event

| SETTING: outpatient, in patient, ED, home, nursing home, rehab, group, etc. | Parent of child who is undergoing a procedure in the operating room. SP is in a private waiting room outside of the operating room. |
| --- | --- |
| PATIENT PROFILE: Information about the “patient” that helps select an SP and helps the learner get an understanding of them as a person. SP will know more information about the patient than learner will ever ask but allows SP to portray a fully developed patient personality. If none of the items below are particulars for the case, please write “all may be used.” | |
| Age range | 30-40 years old |
| Religious/spiritual background | All may be used |
| Sex (e.g., male, female, intersex, transwoman, transman) | All may be used |
| Sexual orientation (e.g., heterosexual, lesbian, gay, bisexual, pansexual, queer, asexual) | All may be used |
| Gender expression (e.g., man, woman, genderqueer) | All may be used |
| Race and ethnicity | All may be used |
| Physical description (e.g., BMI, height range) | All may be used |
| Physical limitations | none |
| Patient appearance (e.g., disheveled, hospital gown, business casual, casual) | Casual but tidy, wearing comfortable clothing suitable for a long day at the hospital. |
| Moulage + location (e.g., none, bruises, scars, body piercing, tattoos) | none |
| Affect (e.g., pleasant, cooperative) | pleasant, but nervous |
| Family group (e.g., who is family, who they live with) | Lives in city with partner and two children |
| Education | Some College, did not graduate |
| Level of health literacy | Medium |
| Employment, if any - present and past, noting any current stresses | Sales Executive |
| Home/homeless - type of dwelling, number of stories, owned or rented | Apartment in city |
| Financial situation - any current stresses | no financial stresses |
| Insurance status (e.g., un/under/insured, public/private, HMO/PPO) | private |
| Habits (i.e., diet, exercise, caffeine, smoking, alcohol, drugs) | None |
| Activities (i.e., hobbies, sports, clubs, friends) | All may be used |
| Typical day - what is the usual daily routine | goes to work daily, spends time with partner (if applicable) and children when at home |

| CASE INFORMATION | |
| --- | --- |
| Chief Concern: What the patient will say when greeted by the student. The patient’s primary reason for seeking medical care often stated in their own words. | I’m waiting for an update about my child’s planned procedure they underwent today |
| Additional Concerns: Other, if any, concerns the patient has today (i.e., symptoms, requests, expectations, etc.) that will become part of set agenda. | Is their child going to recover from this procedure? How can the mistake be avoided again? Will they have to pay for the prolonged case? |
| THE PATIENT’S STORY: The SP will be asked to tell their symptom story and the personal and emotion impact for each of their concerns. You will want to write this in the patient’s voice. The symptom story should be able to answer this question: “Tell me more about [chief concern/additional concern], starting at the beginning and bringing me up to now.”  The personal context should be able to answer questions concerning the broader personal/psychosocial context of symptoms, especially the patient’s beliefs/attributions.  The emotional context should be able to ask how are you doing with this, how does this make you feel, how has this affected you emotionally? IMPACT: How has this affected your life? How has this been for your family? | I can’t believe what a day we are having. We were riding bikes after lunch and my 7-year-old son Carson fell and broke his arm. We came to the emergency room immediately after and was told that he needed to have surgery. Surgery has been going for two hours, and I am anxious to hear how my son is doing. Carson has always been very healthy and has never had surgery before.  I’m glad you’re the anesthesia practitioner is here to update me about my son Carson’s surgery. Obviously, when your child needs a surgery, it can be nerve-wracking. He’s been in the surgery for a couple of hours now, so I just want to hear how he’s doing.  I am upset to find out that one of the medications that Carson is allergic to has been given to him. So many questions are running through my mind: “How did this happen?”, “Is he going to be okay?”, “Can I see him/her”, “How can I explain this to my spouse?”.  I’m relieved to find out that there should be no long-term effects from the medication error, but I want to make sure this never happens again. Most of all, I just want to see my son. |
| HISTORY OF PRESENT ILLNESS: Although some of the HPI will be given in the patient’s symptom story, the learners will expand the story during the direct question section. Below, describes the detailed history, usually about the chief concern, which the student must develop in order to make a useful assessment of the problem: | |
| Onset (when; gradual or sudden) | Not applicable |
| Setting (what was going on or where was patient when symptoms first noticed?) | SP’s child experienced an intraoperative adverse event |
| Duration (how long) | SP has been in the waiting room since the procedure started two hours ago |
| Time relationships (frequency, constant or intermittent) | Not applicable. |
| Location | Not applicable |
| Radiation | Not applicable |
| Quality | Not applicable |
| Amount | Not applicable |
| Aggravated by what | Not applicable |
| Relieved by what | Not applicable |
| Associated with what | Not applicable |
| Attitude (what does the patient think is the problem, and how do they feel about it) | The SP has anxiety about their child needing a surgery. They display a frustrated but reasonable tone after news of adverse medication administration. The are worried about their son’s outcome. |
| Overall course | The pediatric patient in the operating room will completely recover from the complication without any ill effects. The child will need to spend slightly more time in the hospital for monitoring |
| REVIEW OF SYSTEMS: Significant positives and negatives | |
| Constitutional - not applicable | Genito-urinary - not applicable |
| HEENT – not applicable | Musculoskeletal - not applicable |
| Cardiovascular – not applicable | Skin/breast -not applicable |
| Respiratory - not applicable | Neurological - not applicable |
| Gastroenterology - not applicable | Psychiatric - not applicable |
| Past medical history |  |
| Medication allergies (name and reaction) | not applicable |
| Environmental allergies (name and reaction) | not applicable |
| Illnesses | not applicable |
| Vaccinations | not applicable |
| Surgeries | not applicable |
| Accidents/injuries/trauma | not applicable |
| Hospitalization | not applicable |
|  | |
| Inclusive sexual and reproductive history | |
| Sexual practices  Sexual partners  Protection: Use of safer sex practices  Use of birth control if appropriate  Risk of intimate partner violence | not applicable |
| OB/GYN history | Age of onset of menses: not applicable  Age of menopause: not applicable  Number of pregnancies: not applicable  Number of live births: not applicable  Number of miscarriages: not applicable  Number of abortions: not applicable |
| Medications | not applicable |
| Immunizations not applicable | X Tetanus  X Flu  X Hepatitis  X Pneumovax  X HPV  X COVID |
| Tobacco products: not applicable   - Cigarettes - Cigar - Pipe - Chew - E-cigarettes | X Never   - Past - year started/year quit - Current   - Quantity   - # of years |
| Alcohol not applicable   - Beer - Wine - Liquor - Other | X Never   - Past - year started/year quit - Current   - Quantity   - # of years |
| Drugs not applicable   - Weed - Cocaine - Heroin - Meth - IV - Inhalants - Other | X Never   - Past - year started/year quit - Current   - Quantity   - # of years |
| Diet (describe) | not applicable |
| Exercise (describe) | not applicable |
| List any other important social history or information important to this case | Not applicable |
| Family history | not applicable |
| Mother, father, siblings, grandparents, and other significant findings | not applicable |
|  |  |
| Physical Exam – Not applicable | |
| PHYSICAL EXAM FINDINGS |  |
| 1. Written in layperson’s terms | Not applicable |
| 1. General appearance - affect, appearance, position of patient at opening (i.e., sitting, lying down, holding abdomen, etc.) | Not applicable |
| 1. Vital signs | Not applicable |
| 1. Specific findings and affect | Not applicable |
| 1. Response to certain physical movements | Not applicable |
|  |  |
| DIAGNOSIS AND DIFFERENTIAL |  |
| Diagnosis with support from positive and negative history and PE findings | Not applicable |
| Differential with support from positive and negative history and PE findings | Not applicable |
|  |  |
| MANAGEMENT OR DIAGNOSTIC PLAN | Anesthesia practitioner must inform SP that their son had an allergic reaction after being given the wrong antibiotic for surgical prophylaxis in the operating room. |
|  |  |
| PROFESSIONALISM ISSUES OR CHALLENGES | Adverse event regarding wrong medication administration; Breaking bad news |
