# Supplementary material for: Perioperative Difficult Conversations With Guardians of Pediatric Patients: A Simulation-Based Workshop for Anesthesiology Practitioners Using the VitalTalk Framework
Source: MedEdPORTAL. 2026 Jul 7;22:11616. doi: 10.15766/mep_2374-8265.11616 (PMC13337673; doi:10.15766/mep_2374-8265.11616)
Supplement: Supplementary file 1 — SP Handout.docxLearner Case Stems.docxSP Case for Pretest.docxSlide Deck Didactic.pptxDeliberate Practice 1 Scenario.docxDeliberate Practice 2 Scenario.docxChecklist.docxSP Case for Posttest.docxSP Case for Delayed Posttest.docxPost Course Survey.docx [file mep_2374-8265.11616-s001.zip › I. SP Case for Delayed Posttest.docx]

Appendix I: Delayed Post-test Encounter *MedEdPORTAL* Standardized Patient Case Development Tool

This appendix contains detailed case information for the facilitator for the Delayed Post-test encounter.

Date: December 9^th^, 2024

Primary Case Author: Heather Ballard MD, MS

Secondary Case Author: Mitchell Phillips MD

Standardized Patient Educator: Mitchell Phillips MD

Name of Case: Perioperative Difficult Conversations: A Simulated Patient Case Workshop for Anesthesiology Practitioners

Name of Educational and/or Assessment Activity: Adverse event- wrong medication administration

Parent Name: Juan/Juanita Rodriguez (Child: Alexa Rodriguez)

Chief Complaint: Seeking information after daughter is given medication that can affect their daughter’s kidney disease

Most Likely Diagnosis and Differential With Rationale From History and/or Physical Exam: Not applicable

Challenge Question(s):

How could this mistake happen when it’s clearly documented in my child’s medical record?

Is my child going to be okay?

What are the immediate and long-term effects on my child’s kidneys

What steps are you taking to monitor and protect my child now?

Who is responsible for this error?

Domains: Check all that apply

X Professionalism

X Communication and Interpersonal Skills

Medical History

Physical Exam

Shared Decision-Making

X Patient Education

Clinical Reasoning

Documentation

Handoff

Presentation

Other:

Type and Level of Learner: Anesthesiology practitioners: Attending Anesthesiologists and Certified Registered Nurse Anesthetists

Case Objectives: Please list specific objectives for each of the domains you have checked above:

1. Apply NURSE (naming, understanding, respecting, supporting, exploring) framework to respond to SP’s emotions with empathy and professionalism
2. Apply SPIKES (setting, perception, invitation, knowledge, emotion, summary/next steps) framework to communicate with SP about child’s adverse event (allergic reaction).
3. Demonstrate SP’s understanding of child’s wrong medication administration through education surrounding medical details of adverse event

| SETTING: outpatient, in patient, ED, home, nursing home, rehab, group, etc. | Parent of child who is undergoing a procedure in the operating room. SP is in a private waiting room outside of the operating room. |
| --- | --- |
| PATIENT PROFILE: Information about the “patient” that helps select an SP and helps the learner get an understanding of them as a person. SP will know more information about the patient than learner will ever ask but allows SP to portray a fully developed patient personality. If none of the items below are particulars for the case, please write “all may be used.” | |
| Age range | 30-40 years old |
| Religious/spiritual background | All may be used |
| Sex (e.g., male, female, intersex, transwoman, transman) | All may be used |
| Sexual orientation (e.g., heterosexual, lesbian, gay, bisexual, pansexual, queer, asexual) | All may be used |
| Gender expression (e.g., man, woman, genderqueer) | All may be used |
| Race and ethnicity | All may be used |
| Physical description (e.g., BMI, height range) | All may be used |
| Physical limitations | none |
| Patient appearance (e.g., disheveled, hospital gown, business casual, casual) | Business casual attire |
| Moulage + location (e.g., none, bruises, scars, body piercing, tattoos) | none |
| Affect (e.g., pleasant, cooperative) | Shows a combination of worry and protectiveness. When interacting with the physician, parent’s behavior is attentive and cautious, with a tone that conveys deep concern and need for reassurance. |
| Family group (e.g., who is family, who they live with) | Lives in city with partner and two children |
| Education | College |
| Level of health literacy | High |
| Employment, if any - present and past, noting any current stresses | Full-time professional |
| Home/homeless - type of dwelling, number of stories, owned or rented | Townhouse in city |
| Financial situation - any current stresses | no financial stresses |
| Insurance status (e.g., un/under/insured, public/private, HMO/PPO) | private |
| Habits (i.e., diet, exercise, caffeine, smoking, alcohol, drugs) | None |
| Activities (i.e., hobbies, sports, clubs, friends) | All may be used |
| Typical day - what is the usual daily routine | goes to work daily, spends time with partner (if applicable) and children when at home |

| CASE INFORMATION | |
| --- | --- |
| Chief Concern: What the patient will say when greeted by the student. The patient’s primary reason for seeking medical care often stated in their own words. | I’m waiting for updates after my child has undergone emergency appendectomy. |
| Additional Concerns: Other, if any, concerns the patient has today (i.e., symptoms, requests, expectations, etc.) that will become part of set agenda. | How could this mistake happen when it’s clearly documented in my child’s medical record?  Is my child going to be okay?  What are the immediate and long-term effects on my child’s kidneys  What steps are you taking to monitor and protect my child now?  Who is responsible for this error? |
| THE PATIENT’S STORY: The SP will be asked to tell their symptom story and the personal and emotion impact for each of their concerns. You will want to write this in the patient’s voice. The symptom story should be able to answer this question: “Tell me more about [chief concern/additional concern], starting at the beginning and bringing me up to now.”  The personal context should be able to answer questions concerning the broader personal/psychosocial context of symptoms, especially the patient’s beliefs/attributions.  The emotional context should be able to ask how are you doing with this, how does this make you feel, how has this affected you emotionally? IMPACT: How has this affected your life? How has this been for your family? | I’m in the surgical waiting room, waiting for updates about my daughter Alexa. I came straight from work after I found out that she needed to have emergency surgery to remove her appendix. I thought it was just indigestion from last night’s dinner. I’m anxious to hear how the surgery went and doubly nervous because Alexa has chronic kidney disease, so we see doctors every couple of months to check on her.  When I found out that Alexa received a medication that could affect her kidney function, first I am in shock. How could that happen in a hospital like this? Who made this mistake? And then I am angry that this medicine could affect her kidneys in the long term. What is going to happen to her? How can we make sure it doesn’t happen again? |
| HISTORY OF PRESENT ILLNESS: Although some of the HPI will be given in the patient’s symptom story, the learners will expand the story during the direct question section. Below, describes the detailed history, usually about the chief concern, which the student must develop in order to make a useful assessment of the problem: | |
| Onset (when; gradual or sudden) | Not applicable |
| Setting (what was going on or where was patient when symptoms first noticed?) | SP’s child experienced an intraoperative adverse event |
| Duration (how long) | SP has been in the waiting room since the procedure started an hour ago |
| Time relationships (frequency, constant or intermittent) | Not applicable. |
| Location | Not applicable |
| Radiation | Not applicable |
| Quality | Not applicable |
| Amount | Not applicable |
| Aggravated by what | Not applicable |
| Relieved by what | Not applicable |
| Associated with what | Not applicable |
| Attitude (what does the patient think is the problem, and how do they feel about it) | The SP has anxiety about their child needing a surgery. They display a frustrated but reasonable tone after news of adverse medication administration. The are worried about their daughter’s kidney function. |
| Overall course | The SP’s daughter will need monitoring of their kidney function, but will not have any long-term adverse effects |
| REVIEW OF SYSTEMS: Significant positives and negatives | |
| Constitutional - not applicable | Genito-urinary - not applicable |
| HEENT –not applicable | Musculoskeletal - not applicable |
| Cardiovascular – not applicable | Skin/breast - not applicable |
| Respiratory - not applicable | Neurological - not applicable |
| Gastroenterology - not applicable | Psychiatric - not applicable |
| Past medical history |  |
| Medication allergies (name and reaction) | not applicable |
| Environmental allergies (name and reaction) | not applicable |
| Illnesses | not applicable |
| Vaccinations | not applicable |
| Surgeries | not applicable |
| Accidents/injuries/trauma | not applicable |
| Hospitalization | not applicable |
|  | |
| Inclusive sexual and reproductive history | |
| Sexual practices  Sexual partners  Protection: Use of safer sex practices  Use of birth control if appropriate  Risk of intimate partner violence | not applicable |
| OB/GYN history | Age of onset of menses: not applicable  Age of menopause: not applicable  Number of pregnancies: not applicable  Number of live births: not applicable  Number of miscarriages: not applicable  Number of abortions: not applicable |
| Medications | not applicable |
| Immunizations not applicable | X Tetanus  X Flu  X Hepatitis  X Pneumovax  X HPV  X COVID |
| Tobacco products: not applicable   - Cigarettes - Cigar - Pipe - Chew - E-cigarettes | X Never   - Past - year started/year quit - Current   - Quantity   - # of years |
| Alcohol not applicable   - Beer - Wine - Liquor - Other | X Never   - Past - year started/year quit - Current   - Quantity   - # of years |
| Drugs not applicable   - Weed - Cocaine - Heroin - Meth - IV - Inhalants - Other | X Never   - Past - year started/year quit - Current   - Quantity   - # of years |
| Diet (describe) | not applicable |
| Exercise (describe) | not applicable |
| List any other important social history or information important to this case | Not applicable |
| Family history | not applicable |
| Mother, father, siblings, grandparents, and other significant findings | not applicable |
|  |  |
| Physical Exam – Not applicable | |
| PHYSICAL EXAM FINDINGS |  |
| 1. Written in layperson’s terms | Not applicable |
| 1. General appearance - affect, appearance, position of patient at opening (i.e., sitting, lying down, holding abdomen, etc.) | Not applicable |
| 1. Vital signs | Not applicable |
| 1. Specific findings and affect | Not applicable |
| 1. Response to certain physical movements | Not applicable |
|  |  |
| DIAGNOSIS AND DIFFERENTIAL |  |
| Diagnosis with support from positive and negative history and PE findings | Not applicable |
| Differential with support from positive and negative history and PE findings | Not applicable |
|  |  |
| MANAGEMENT OR DIAGNOSTIC PLAN | Anesthesia practitioner must inform SP that their daughter was given a medication that could exacerbate her chronic kidney disease. |
|  |  |
| PROFESSIONALISM ISSUES OR CHALLENGES | Adverse event regarding wrong medication administration; Breaking bad news |
